# Neurogenesis-independent mechanisms of MRI-detectable hippocampal volume increase following electroconvulsive stimulation

**DOI:** 10.1038/s41386-023-01791-1

**Published:** 2024-01-09

**Authors:** Yoshifumi Abe, Kiichi Yokoyama, Tomonobu Kato, Sho Yagishita, Kenji F. Tanaka, Akihiro Takamiya

**Affiliations:** 1 Division of Brain Sciences, Institute for Advanced Medical Research, Keio University School of Medicine, Tokyo, 35 Shinanomachi, Tokyo, Shinju-ku 160-8582 Japan; 2 Laboratory of Structural Physiology, Center for Disease Biology and Integrative Medicine, Faculty of Medicine, The University of Tokyo, 7-3-1 Hongo, Bunkyo-ku, Tokyo, 113-0033 Japan; 3 Neuropsychiatry, Department of Neurosciences, Leuven Brain Institute, KU Leuven, Leuven, Belgium; 4 Department of Neuropsychiatry, Keio University School of Medicine, Tokyo, Japan; 5 Hills Joint Research Laboratory for Future Preventive Medicine and Wellness, Keio University School of Medicine, Tokyo, Japan

**Keywords:** Cellular neuroscience, Brain

## Abstract

Electroconvulsive therapy (ECT) is one of the most effective psychiatric treatments but the underlying mechanisms are still unclear. In vivo human magnetic resonance imaging (MRI) studies have consistently reported ECT-induced transient hippocampal volume increases, and an animal model of ECT (electroconvulsive stimulation: ECS) was shown to increase neurogenesis. However, a causal relationship between neurogenesis and MRI-detectable hippocampal volume increases following ECT has not been verified. In this study, mice were randomly allocated into four groups, each undergoing a different number of ECS sessions (e.g., 0, 3, 6, 9). T2-weighted images were acquired using 11.7-tesla MRI. A whole brain voxel-based morphometry analysis was conducted to identify any ECS-induced brain volume changes. Additionally, a histological examination with super-resolution microscopy was conducted to investigate microstructural changes in the brain regions that showed volume changes following ECS. Furthermore, parallel experiments were performed on X-ray-irradiated mice to investigate the causal relationship between neurogenesis and ECS-related volume changes. As a result, we revealed for the first time that ECS induced MRI-detectable, dose-dependent hippocampal volume increase in mice. Furthermore, increased hippocampal volumes following ECS were seen even in mice lacking neurogenesis, suggesting that neurogenesis is not required for the increase. The comprehensive histological analyses identified an increase in excitatory synaptic density in the ventral CA1 as the major contributor to the observed hippocampal volume increase following ECS. Our findings demonstrate that modification of synaptic structures rather than neurogenesis may be the underlying biological mechanism of ECT/ECS-induced hippocampal volume increase.

## Introduction

Electroconvulsive therapy (ECT) is one of the most effective psychiatric treatments for acute psychiatric symptoms. For instance, ECT is effective for patients with depression [1], bipolar disorder [2, 3], and schizophrenia [4], who do not respond to other treatments, including pharmacotherapy. Thus, a better understanding of the mechanisms underlying ECT efficacy may provide some direction towards the development of new treatments for severe pharmacotherapy-resistant psychiatric disorders.

Neuroplasticity refers to the brain’s ability to reorganize neural networks, which can be observed at molecular and cellular levels up through large-scale brain networks. Both human and animal studies have suggested that neuroplastic change in the hippocampus is a shared mechanism for several psychiatric treatments [5]. In vivo human neuroimaging studies have consistently reported hippocampal volume increases following ECT [6, 7], which is consistent with the neuroplastic hypothesis for the mechanisms underlying ECT [8]. Importantly, the observed hippocampal volume increases could not be explained only by edematous change [9–11]. Whilst the relationship between the hippocampal volume changes and antidepressant effects remains under debate [7, 12, 13], several ECT parameters, such as the number of sessions [7, 12], the effect of electrical stimulation [13], and induced seizure [14], were associated with hippocampal volume increase following ECT as detected by magnetic resonance imaging (MRI). The increased volume returned to the baseline levels within three to six months [15, 16], and might be reflective of a short window for structural and/or functional network remodeling [17]. Consistent with this idea, a hippocampal subfield analyses revealed that the largest effect of ECT on the hippocampus was observed in the dentate gyrus (DG) [16, 18, 19], where neurogenesis in known to occur.

Electroconvulsive stimulation (ECS), an animal model of ECT, was shown to manifest an increased number of newborn cells in the subgranular zone of the DG in rodents [20, 21], and nonhuman primates [22]. Additionally, ECS-induced antidepressant-like behavioral changes were not observed in a pharmacogenetic rodent model lacking adult neurogenesis [23], suggesting that ECS requires hippocampal neurogenesis to exert an antidepressant-like effect. Similarly, some behavioral effects of antidepressant medications (e.g., fluoxetine) were blocked when neurogenesis was inhibited [24, 25], and increasing hippocampal neurogenesis reduced depression- and anxiety-like behaviors [26]. Considering all the evidence from human neuroimaging and preclinical histological studies, one may speculate that the MRI-detectable hippocampal volume increases following ECT could be induced by neuroplastic cellular changes, particularly neurogenesis. However, the results from previous preclinical histological studies cannot be directly translated or associated with the results of human MRI studies. To address this issue, preclinical evidence of a causal relationship between neurogenesis and MRI-detectable hippocampal volume increases following ECS is needed.

Here, we applied a reverse-translational approach to investigate the role of neurogenesis in the context of hippocampal volume increases measured by MRI. Our research questions were as follows: 1) Is neurogenesis required for MRI-detectable hippocampal volume increases following ECS, and 2) If neurogenesis is not required for volumetric changes, which microstructural changes may contribute to the MRI-detectable changes in volume? In this study, we first confirmed with MRI that ECS resulted in increased hippocampal volumes. Then, we repeated the same experiment using X-ray-irradiated mice, where neurogenesis was ablated, to show a causal relationship between neurogenesis and MRI-detectable hippocampal volume increases following ECS. Following these experiments, we conducted a comprehensive histological analysis to detect the microstructural changes, which were associated with the hippocampal volume increases.

## Methods

A detailed description of the methods can be found in the Supplementary Methods. All animal procedures were conducted in accordance with the National Institutes of Health Guide for the Care and Use of Laboratory Animals and were approved by the Animal Research Committee of Keio University School of Medicine (21031). Experiments were performed using 8-week-old male C57BL/6j mice, which were purchased from Oriental Yeast Co., Ltd. (Tokyo, Japan). ECS was administered to mice once daily, three times a week. ECS was given via bilateral ear clip electrodes with 25 mA and 0.5 msec pulse width for 1 second at a frequency of 100 Hz square wave pulses (UgoBasile, Comerio, Italy). Our histological analysis investigated the following microstructures with antibodies: neurogenesis (anti-DCX), excitatory terminal (anti-VGluT1), excitatory spine (anti-PSD95), inhibitory terminal (anti-VGAT), PV interneuron (anti-PV), myelin (anti-PLP), neuronal soma (anti-NeuN), astrocyte (anti-GLT1), and microglia (anti-Iba1). These microstructural changes were analyzed using super-resolution microscopy (SRM).

## Results

### ECS induces MRI-detectable hippocampal volume increases

**Fig. 1:**
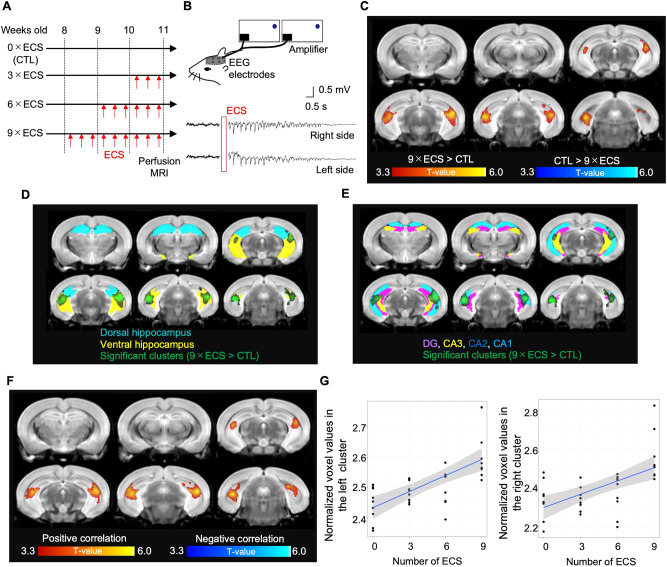
ECS induces hippocampal volume increases as measured by MRI. **A** The time course of ECS. **B** The EEG was recorded bilaterally during the session of ECS. **C** A whole brain voxel-wise group comparison between CTL (0×ECS; *n* = 12) and 9×ECS (*n* = 12). The red color represents the volume increases while the blue color represents the volume decreases. **D** The significant clusters (green: 9×ECS > CTL) were overlayed on the identified dorsal (cyan) and ventral (yellow) hippocampal regions. **E** The significant clusters (green: 9×ECS > CTL) were overlayed on the identified DG (purple), CA3 (yellow), CA2 (blue), and CA1 (cyan). **F** Results of the whole brain voxel-wise regression analysis, including the gray matter volume as a dependent variable, the number of ECS sessions as an independent variable, and the TBV as a nuisance covariate. The red color represents a significant positive correlation while the blue color represents a significant negative correlation. **G** Scatter plots of normalized voxel values (i.e., divided by the TBV and then multiplied by 1000) in the left and right clusters identified by the whole brain regression analysis. There were significant correlations of the number of ECS sessions with the left (*r* = 0.71, df=46, *p* < 0.001) and right (*r* = 0.63, df=46, *p* < 0.001) hippocampal volumes.

Eight-week-old male C57BL/6j mice were randomly allocated into four groups (*n* = 12 in each group), including sham-ECS (i.e., 0×ECS), 3×ECS, 6×ECS, and 9×ECS, to investigate 1) if 9×ECS induced MRI-detectable changes in brain volumes (i.e., a group comparison between sham-ECS and 9×ECS), and 2) whether the ECS-induced brain volume changes were dose-dependent (i.e., a regression analysis using data from the four groups) (Fig. 1A). An ex vivo MRI assessment was conducted after the last ECS session. Our ECS protocol successfully induced generalized seizures, which were confirmed by electroencephalography (EEG) (Fig. 1B).

Whole-brain voxel-wise analysis of MRI data revealed that 9×ECS increased bilateral ventral hippocampal volumes, particularly in the CA1 and DG, and did not result in any decreased brain volumes (Fig. 1C–E, and Supplementary Fig. 1A). The distinction between ventral and dorsal was defined by the gene expression pattern of the wolframin ER transmembrane glycoprotein (Wfs1) [27] (Supplementary Fig 1B). Hippocampal subfields were defined by the Allen Brain Atlas [28]. The identified clusters mainly included the CA1 (left: 42% in total voxel size of the cluster; right: 45%) and DG (left: 12%; right: 11%), but also included the CA2 (left: 0.9%; right: 0.7%) and the CA3 (left: 6%; right: 4%) (Fig. 1E). The results of the region-of-interest (ROI)-based analysis are provided in Supplementary Table 1.

Because in vivo human neuroimaging studies have reported a positive correlation between the number of ECT sessions and the hippocampal volume increase [7], we also investigated the effect of the number of ECS sessions on MRI-detectable hippocampal volume changes. A whole brain regression analysis, including whole brain gray matter volume (GMV) as a dependent variable, the number of ECS sessions as an independent variable, and the total brain volume (TBV) as a nuisance covariate, revealed that two clusters located in the bilateral hippocampal volumes showed significant positive correlations with the number of ECS sessions (left hippocampus: *r* = 0.71, df=46, *p* < 0.001; right hippocampus: *r* = 0.63, df=46, *p* < 0.001) (Fig. 1F, G). These results are consistent with the findings from human ECT-MRI studies, suggesting that the results from this ECS-MRI study could be comparable to the results from human studies.

### Neurogenesis is not required for the ECS-induced hippocampal volume increases

**Fig. 2:**
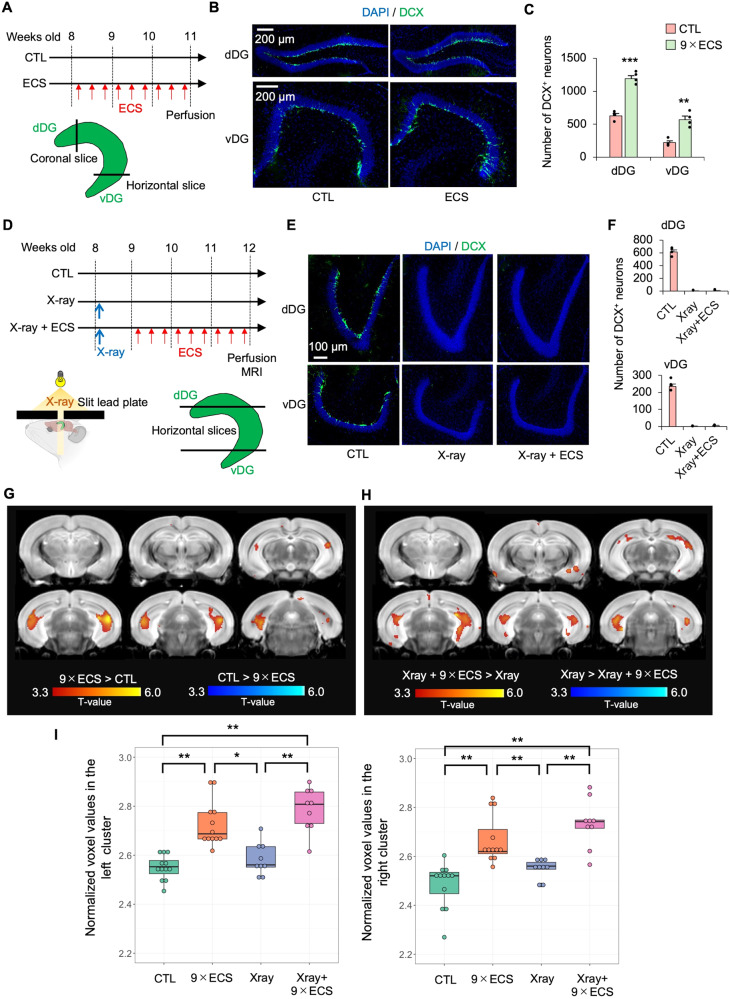
Neurogenesis is not required for ECS-induced hippocampal volume increase. **A** The time course of ECS. The number of the newborn neurons was counted on the hippocampal coronal slices for dDG and horizontal slices for vDG. **B** Representative images of DCX staining in the dDG and vDG. **C** The numbers of DCX^+^ newborn neurons in the dDG and vDG were compared between CTL (*n* = 4) and ECS (*n* = 4). **p < 0.05, ***p < 0.01 (Student’s t-test, vs. CTL). **D** The time course of ECS and X-ray irradiation. A lead plate with a 4-mm slit was placed above the mouse head. The slit in the lead plate was placed just above the whole hippocampus. The number of the newborn neurons was counted on the hippocampal horizontal slices of the dDG and vDG. **E** Representative images of DCX staining in the dDG and vDG. **F** The number of DCX^+^ newborn neurons in the dDG and vDG were compared among CTL (*n* = 4), X-ray (*n* = 4) and X-ray+ECS (*n* = 4). A whole brain voxel-wise group comparison between X-ray (*n* = 12) and X-ray+ECS (*n* = 12) (**G**), and between CTL (*n* = 12) and 9×ECS (*n* = 12) (**H**). The red color represents the volume increases while the blue color represents the volume decreases. **I** Scatter plots of normalized voxel values in the left and right overlapping regions between Figures G and H. A significant main effect of group was observed on both sides (Left: F_3,38_ = 22.6, *p* < 0.001; Right: F_3,38_ = 17.0, *p* < 0.001). The graphs display the results of post-hoc Tukey’s tests (*p < 0.05, **p < 0.01).

A histological analysis confirmed that the ECS protocol significantly increased the number of doublecortin-positive (DCX^+^: a marker of newborn neurons) cells in the dorsal and ventral DG (Fig. 2A–C). To investigate the causal relationship between ECS-induced neurogenesis and MRI-detectable hippocampal volume increases (Fig. 1C), we conducted the same whole-brain analysis using X-ray-irradiated mice (*n* = 24; 12 received 9×ECS and 12 did not). We confirmed that X-ray irradiation markedly decreased the number of DCX^+^ cells in the DG, and that the 9×ECS sessions did not increase the number of DCX^+^ cells in the DG (Fig. 2D–F). These results indicate that the X-ray irradiation protocol successfully ablated neurogenesis in the DG.

Contrary to our expectations, even in the X-ray-irradiated mice, the whole brain MRI analysis identified two clusters showing increased brain volumes following 9×ECS. These clusters mainly included the bilateral hippocampi, particularly the CA1 (left: 34% in total voxel size of the cluster; right: 56%) and the DG (left: 27%; right: 23%), but also included the CA2 (left: 0.3%; right: 0.5%) and the CA3 (left: 4%; right: 7%) (Fig. 2H). The identified brain regions in the analysis were highly overlapped in mice, both with and without X-ray irradiation (Fig. 2G, H). Analyses of the overlapped brain regions revealed that the effect of 9×ECS on the GMV was similar regardless of X-ray irradiation. An ROI-based analysis of the detected clusters also showed that the hippocampal volume was increased by 9×ECS independent of the X-ray irradiation (Fig. 2I and Supplementary Table 1). Regarding the effect of X-ray irradiation on brain volumes, we found that it reduced total brain volumes (Supplementary Fig. 2B), and several brain regions other than the hippocampus (Supplementary Fig. 2C).

Overall, our results indicate that 1) ECS induces MRI-detectable hippocampal volume increases in mice, which is consistent with findings from human MRI studies, and 2) ECS-induced increased neurogenesis is not required for ECS-induced, MRI-detectable hippocampal volume changes.

### RNAseq screening indicates ECS-induced microstructural changes

**Fig. 3:**
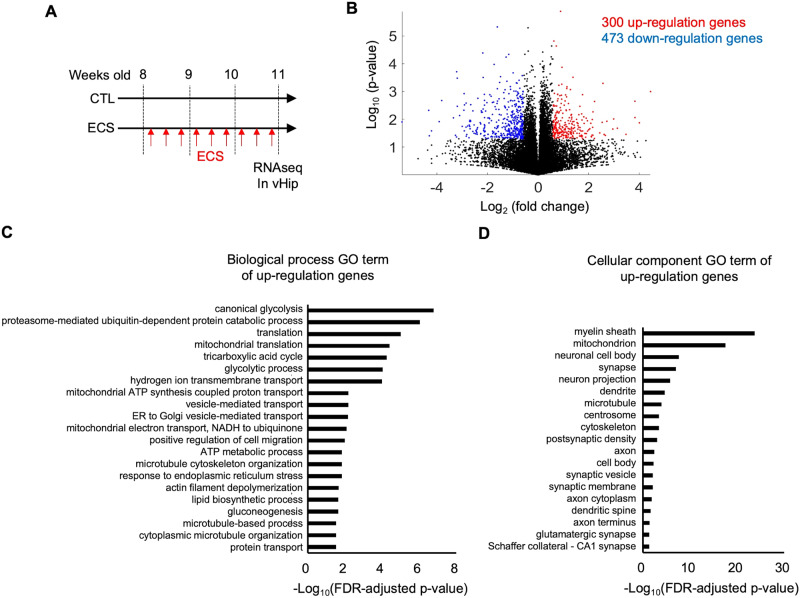
ECS increases the expression level of neuronal microstructure-related genes. **A** The time course of ECS and RNAseq. **B** A volcano plot of RNAseq data. The red color represents significant increases in gene expression levels, while the blue color represents decreases (*p* < 0.05, fold change>1.5). **C**, **D** Significant GO terms for biological processes and cellular components of the upregulated genes. There were no significant GO terms for down regulated genes.

To explore the cellular mechanisms of the MRI-detectable, ECS-induced hippocampal volume increases, we first conducted RNA sequencing (RNAseq) of the 9×ECS-treated hippocampal tissues (Fig. 3A). Based on the results of the whole-brain MRI analysis (Fig. 1C), we focused on the ventral hippocampus. A total of 300 mRNAs were upregulated and 473 were down regulated (Fig. 3B). Using these differential gene expressions (DGEs), we conducted a gene ontology (GO) analysis to examine the associated characteristics. The top significant GO terms from the analysis of the upregulated genes included biological processes related to metabolism, cytoskeleton organization, and cell migration (Fig. 3C). Furthermore, within those involving cellular structure, the top significant GO terms of the upregulated genes involved cellular components related to neuronal microstructure, including the myelin sheath, mitochondria, neuronal cell body, synapses, neuron projections, dendrites, and microtubules (Fig. 3D). In contrast, there were no significant GO terms associated with the down regulated genes. We also investigated changes in the expression of each gene in the hippocampus with ECS (Supplemental Fig. 3). We found that the expression levels of axon-related (e.g., *Tubb3* and *Nefm*), dendrite-related (e.g., *Map2*), and synapse-related (e.g., *Sv2a*, *Syn1*, *Syn2*, and *Nlgn2*) genes were increased, indicating ECS-induced, neuronal microstructural changes. In addition, an investigation of the immediate early genes (e.g., *fos*, *Arc*, *Npas4*, and *Erg1*) showed a reduction in their expressions following ECS, suggesting that 9×ECS might decrease neuronal activity in the hippocampus. In accordance with these results from the RNAseq screening, further histological analyses focused on neuronal microstructural components including axons, dendrites, synapses, and myelin.

### ECS increases dendritic branching in the ventral CA1

**Fig. 4:**
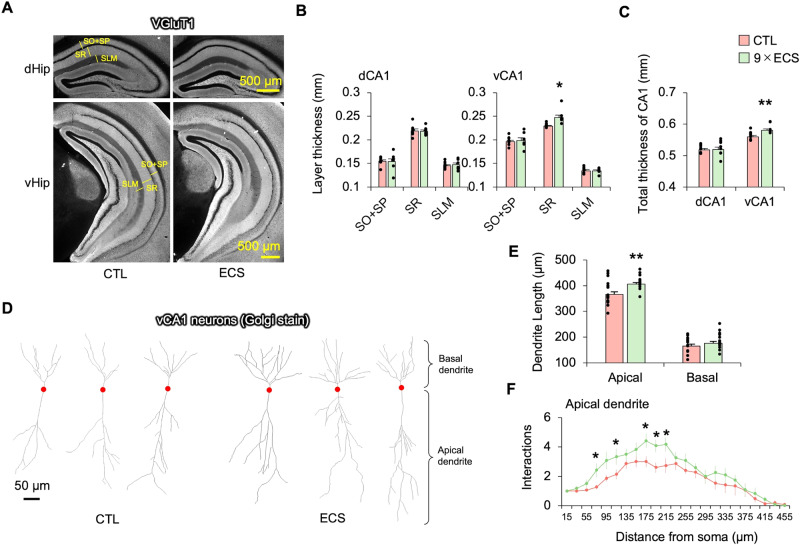
ECS increases the length of the stratum radiatum layer in the vCA1. **A** Representative VGluT1 staining in the dHip and vHip. SO: stratum oriens; SP: stratum pyramidale; SR: stratum radiatum, SLM: stratum lacunosum moleculare. **B**, **C** Each layer thickness and total layer thickness (SO + SP + SR + SLM) in the dCA1 and vCA1 were compared between CTL (*n* = 8) and ECS (*n* = 8). A two-way repeated ANOVA was performed (dCA1: ECS *p* = 0.95, interaction *p* = 0.091, vCA1: ECS *p* = 0.24, interaction *p* = 0.033, Total length: ECS *p* = 0.078, interaction *p* = 0.094). **D** Representative vCA1 neurons with Golgi staining. The red circles represent the somas of the vCA1 neurons. **E** The length of the apical and basal dendrites of the vCA1 neurons were compared between CTL and ECS (17 neurons were measured from three mice of each group). Two-way repeated ANOVA was performed (ECS *p* = 0.0025, interaction *p* = 0.064). **F** Interaction numbers were plotted in a Sholl analysis of the apical dendrites in the vCA1 neurons. A two-way repeated ANOVA was performed (ECS *p* = 6.5 × 10^−10^, interaction *p* = 0.19). *p < 0.05, **p < 0.01 (Student’s t-test, p-values were Bonferroni corrected, vs. CTL).

We focused on the ventral CA1 (vCA1) in the following histological analyses. First, we investigated the macrostructural changes by measuring the thickness of each layer in the vCA1 and dorsal CA1 (dCA1). The thickness of each layer [stratum oriens (SO) + stratum pyramidale (SP), stratum radiatum (SR), and stratum lacunosum moleculare (SLM)] was defined by the contrast of the vesicular glutamate transporter 1 (VGluT1) immunostaining (Fig. 4A; Supplementary Fig. 8). The analysis showed ECS increased the SR layer thickness in the vCA1 but not in the dCA1 (Fig. 4B, C). Second, because the SR layer is composed mainly of dendrites from CA1 neurons, we examined changes in dendritic length and branching by Golgi staining. We found that ECS increased the total length of the apical dendrites in the vCA1, as well as the number of dendritic branching points (Fig. 4D–F).

### ECS increases excitatory and inhibitory terminals and myelin

**Fig. 5:**
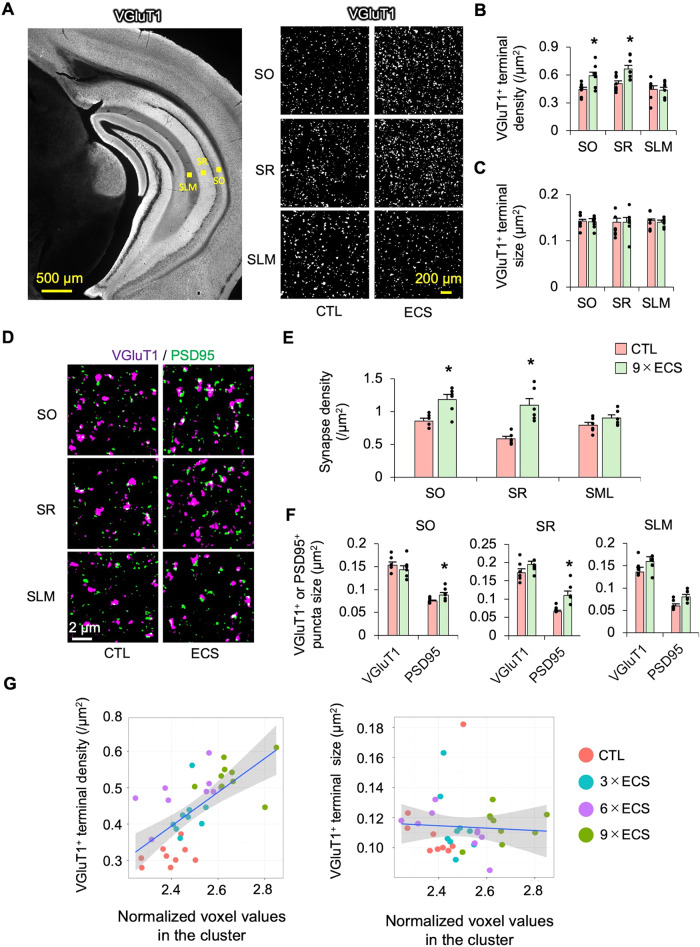
ECS increases excitatory synaptic density. **A** Representative fluorescence microscope image of VGluT1 staining (left) and SRM images (right) in each layer of the vCA1. **B**, **C** The density and size of the VGluT1^+^ excitatory terminals were compared in each layer between CTL (*n* = 8) and ECS (*n* = 8). A two-way repeated ANOVA was performed (Density: ECS *p* <0.001, interaction *p* = 0.031, Size: ECS *p* = 0.77, interaction *p* = 0.97). **D** Representative SRM images of VGluT1 and PSD95 in each layer of the vCA1. **E** The excitatory synaptic density (pairs of VGluT1^+^ puncta and PSD95^+^ puncta) was compared in each layer of the vCA1 between CTL (*n* = 6) and ECS (*n* = 6). A two-way repeated ANOVA was performed (ECS *p* <0.001, interaction *p* = 0.015). **F** The sizes of the pairs of the VGluT1^+^ and PSD95^+^ puncta were compared in each layer of the vCA1 between CTL (*n* = 6) and ECS (*n* = 6). **G** Scatter plots of the density or size of the VGluT1^+^ excitatory terminals in the SR and normalized voxel values in the vCA1. There was a significant correlation of the voxel values in the vCA1 with the VGluT1^+^terminal density (*r* = 0.67, df=34, *p* < 0.001, *R*^2^ = 0.44), but not with VGluT1^+^teminal size (*r* = –0.06, df=34, *p* = 0.73, *R*^2^ = 0.004). The voxel values were normalized using the TBV of each animal, and then the values of the left and right vCA1 were averaged. *p < 0.05 (Student’s t-test, *p* values were Bonferroni corrected, vs. CTL).

Utilizing SRM, we examined the effect of ECS on microstructural components, including excitatory and inhibitory terminals, spines, and myelin. First, ECS increased the density, but not the size, of the VGluT1^+^ excitatory terminals in the SR layer of the vCA1 (Fig. 5A–C). ECS also increased synaptic density (i.e., the number of pairs of VGluT1^+^ puncta and post synaptic density 95 (PSD95)^+^ puncta) and PSD95^+^ spine sizes in the CA1 (Fig. 5D–F). Golgi staining confirmed that ECS increased the spine density and spine head diameter in the SR of the vCA1 neurons (Supplementary Fig. 4; Supplementary Fig. 8C). These results indicate that ECS increased synaptic density in the SR of the vCA1. To investigate the relationship between excitatory synapses and brain volumes measured by MRI, we conducted correlation analyses using MRI and histological measurements in the same animals. Voxel values in the identified clusters from the VBM analysis were normalized to the TBV. Then, the bilateral normalized voxel values were averaged for the subsequent analyses, because the histological analyses did not reveal any indication of laterality. The voxel values in the vCA1 were significantly correlated with the density of the VGluT1^+^ terminals (*r* = 0.67, df=34, *p* < 0.001, *R*^2^ = 0.44), but not with the VGluT1^+^ terminal size (*r* = –0.06, df=34, *p* = 0.73, *R*^2^ = 0.004) (Fig. 5G).

Second, ECS increased the density and the size of the vesicular GABA transporter (VGAT)^+^ inhibitory terminals in the SR layer of the vCA1 (Supplementary Fig. 6A–C; Supplementary Fig. 8D). The voxel values in the vCA1 were significantly correlated with the VGAT^+^ terminal density (*r* = 0.55, df=34, *p* < 0.001, R^2^ = 0.30), and the VGAT^+^ terminal size (*r* = 0.39, df=34, *p* = 0.02, R^2^ = 0.15) (Supplementary Fig. 6D).

Finally, we found that ECS increased the percent area of myelin proteolipid protein (PLP)^+^ in the SR layer of the vCA1 (Supplemental Fig. 7A, B). The diameter of the myelinated axons increased following ECS, yet myelin thickness did not change (Supplemental Fig. 7C, D). Since myelinated axons in the SR mainly originate from inhibitory interneurons including parvalbumin (PV)^+^ interneurons [29], we examined whether myelin in the SR was increased in PV^+^ interneurons. Although ECS did not increase the PV^+^ axon density, it did increase the density of the PV^+^ myelinated axons (i.e., PLP and PV double positive axons), and the ratio of the PV^+^ myelinated axons to all PV^+^ axons (Supplemental Fig. 7E-H), indicating that PV^+^ interneurons are one type of axons with increased myelin following ECS. The voxel values in the vCA1 were significantly correlated with the percent area of the PLP^+^ myelin (*r* = 0.47, df=34, *p* = 0.004, R^2^ = 0.22) (Supplemental Fig. 7I).

A similar histological analysis was performed on the ventral DG, which showed the second most notable volume increase in the cluster identified by the VBM analysis. The synaptic density of the excitatory synapses in the DG decreased, while the inhibitory synapse remained unchanged (Supplementary Fig. 5). All results of the histological analyses are presented in Supplementary Table 2.

Overall, we found that ECS increased the density of excitatory synapses (both spines and terminals), the density and size of the inhibitory terminals, and the percentage of myelin in the SR layer of the vCA1. Among these, the density of the VGluT1^+^ terminals had the highest correlation with the voxel values in the vCA1 (standardized beta coefficient=0.55, *p* = 0.002), suggesting that among the observed microstructural changes, ECS-induced increases in excitatory terminal densities may represent the greatest contribution to the ECS-induced, MRI-detectable hippocampal volume increases. In addition, the number of ECS sessions were positively associated with the density of the excitatory VGluT1^+^ terminals (*r* = 0.85, df=34, *p* < 0.001), inhibitory VGAT^+^ terminals (*r* = 0.65, df=34, *p* < 0.001), and percent area of myelin (*r* = 0.72, df=34, *p* < 0.001) (Supplemental Fig. 9). These results indicate that ECS increased the excitatory and inhibitory synapses as well as myelin in the SR layer of the vCA1 in a dose-dependent manner, similar to the ECT-induced, MRI-detectable, dose-dependent hippocampal volume increases [7].

### ECS increases the density and morphology of microglia but not other glial cells

We examined the effect of ECS on the cellular density and morphology of the vCA1 pyramidal neurons and glial cells. We found that ECS did not change the density of the soma nor the size of the vCA1 pyramidal neurons (Supplemental Fig. 10A, B). In addition, ECS did not change the density of the *Gja1*^+^ astrocytes nor that of the *Plp1*^+^ oligodendrocytes. In contrast, ECS increased the density of *Csf1r*^+^ microglia in the SR layer of the vCA1 (Supplemental Fig. 10C-H). Additionally, ECS increased the percent area of the ionized calcium-binding adaptor molecule 1 (Iba1)^+^ microglia, yet it did not increase the astrocytic glutamate transporter 1 (GLT1)^+^ astrocytes in the SR layer of the vCA1 (Supplemental Fig. 11). These results indicate that ECS increased the volume of activated microglia in the SR.

### ECS induces dendritic arborization, and increases the number of excitatory terminals and myelin in mice lacking neurogenesis

Since our MRI analysis identified hippocampal volume increases following ECS even in X-ray-irradiated mice lacking neurogenesis (Fig. 2G-I), we also conducted histological analyses of the vCA1 in these mice. First, we found that ECS increased the SR layer thickness, the total length of the apical dendrites, and the number of dendritic branches in the vCA1 (Supplemental Fig. 12A–E). Second, we found significant increases in the density of the VGluT1^+^ terminals (t = 2.85, df=10, *p* = 0.02) and the percent area of the PLP^+^ myelin (t = 9.36, df=10, *p* < 0.01), but not in the density of the VGAT^+^ terminals (t = 1.32, df=10, *p* = 0.22) (Supplemental Fig. 12F-H). Golgi staining confirmed that ECS increased spine density and spine head diameter in the SR of the vCA1 (Supplemental Fig. 13). These results indicate that ECS induces dendritic and synaptic changes regardless of the presence of neurogenesis.

In addition, X-ray irradiation itself increased the percent area of the GLT1^+^ astrocytes and Iba1^+^ microglia. However, ECS did not induce additional changes in astrocytes or microglia in the X-ray-irradiated mice (Supplemental Fig. 14). These results suggest that activated astrocytes and microglia do not contribute to the ECS-induced volume increases in the X-ray-irradiated mice.

## Discussion

In the current study, we showed for the first time, that an animal model of ECT induced MRI-detectable hippocampal volume increases in a dose-dependent manner using an unbiased, whole brain, voxel-wise analysis. This result is consistent with findings from human neuroimaging studies [6, 7]. Since previous human studies have reported the effect of ECT was greatest in the DG where neurogenesis occurs [10, 16, 19], and ECS strongly enhances adult neurogenesis in the DG of rodents, we hypothesized that neurogenesis was necessary for the MRI-detectable volume increases. Contrary to our expectations, however, the MRI-detectable hippocampal volume increases following ECS were observed even in mice lacking neurogenesis, suggesting that neurogenesis is not required for the ECS-induced volume increases. Our comprehensive histological analyses indicated that dendritic arborization, synaptic structural modifications, and increased myelin might contribute to the MRI-detectable, dose-dependent hippocampal volume increases following ECS. Our results provide the first evidence of the cellular mechanisms underlying ECS-induced, MRI-detectable, hippocampal volume increase, which may be considered a reasonable interpretation of the findings from human ECT-MRI studies.

In vivo human MRI-measurements (e.g., gray matter volumes) are a valuable tool to noninvasively assess brain structural plasticity. However, combined MRI and histological studies are required to directly link MRI-derived measures with underlying cellular alterations, because MRI-derived measures alone are not specific to particular underlying microstructural components [30]. An important and unexpected finding in the present study is that the MRI-detectable hippocampal volume increases following ECS were observed regardless of the presence of neurogenesis. Histological analysis revealed increased excitatory and inhibitory terminals, as well as myelin in the CA1, which correlated with MRI-measurements (Fig. 5 and Supplemental Fig. 6, 7). Among those microstructural changes, alterations in the density of the excitatory terminals showed the highest correlation with the hippocampal volume increases following ECS. These results suggest that changes in the excitatory terminals were the major contributor to the hippocampal volume increases following ECS.

In addition to the changes in terminals, ECS also increased the number of dendritic branches and spine density, both of which were reported as the basis for MRI-detectable GMV changes [31, 32], and were observed in previous ECS studies [33, 34]. Although it is difficult to identify the cellular component which contributed the most to the volume increase, our results suggest that a modification of synaptic structures (e.g., dendritic spines and axon terminals) induces the ECS-induced hippocampal volume increases in the vCA1. In line with our findings, a previous study reported ECS-induced increases in the number of spines in the CA1 [35]. Because the majority of excitatory synapses are generally formed on dendritic spines, the previous result may suggest that the main effect of ECS was on excitatory synapses. In addition, an increase in the number of spines in the CA1 has also been reported as an effect of antidepressant medications [36, 37], suggesting a shared mechanism for different antidepressant treatments. Morphological changes in dendrites and synapses could alter neurotransmission, synaptic function, and neural plasticity [38]. Hence, future studies should investigate how the histological changes in the CA1 might be related to changes in hippocampal function and antidepressant-like effects. It should be noted that our results did not indicate that the CA1 was the primary target region of ECS because the VGluT1^+^ excitatory terminals in the SR layer of the CA1 were mainly projections from the dorsal and ventral CA3 [39]. Moreover, neurogenesis in the DG could be an upstream event for neuroplastic changes in the CA1 [40, 41]. Therefore, ECS may primarily induce plastic changes in the CA3 pyramidal neurons or neurons in the DG, which are more upstream subnuclei within the hippocampal circuitry.

Although ECS also increased inhibitory terminals, our post-hoc additional analyses suggested a possibility that the VGAT^+^ inhibitory terminals did not increase linearly in a dose-dependent manner, but rather they decreased with less than three ECS sessions and increased with more than six ECS sessions (Supplemental Fig. 9B, C). Our results from additional histological analyses suggest that a small number of ECS sessions enhanced the excitatory input to the vCA1, whereas repeated ECS sessions also enhanced inhibitory input to the vCA1. Each stage of ECS could exhibit differential effects on excitatory and inhibitory input. Future studies should investigate the early and late effects of ECS on the excitatory-inhibitory balance during a course of ECS. Our results from the RNAseq analysis, which showed decreased neural activity markers following 9×ECS sessions, support decreased neural activity in the late stages of ECS.

Our histological analysis revealed for the first time that ECS increased myelin in the vCA1. In addition, the neuronal subtype exhibiting increased myelin was the PV^+^ inhibitory interneuron. Some studies have reported that chronic stress reduces PV^+^ inhibitory interneurons in the hippocampus, particularly in the CA1, CA2/3, and DG, and that antidepressant medications also increased the number of the PV^+^ neurons. However, these findings have been inconsistent [42]. In addition, human postmortem studies reported decreased PV^+^ interneurons in patients with mood disorders [43, 44]. Although it is speculative, our findings of the ECS-induced increases in myelin in the vCA1, might be associated with the underlying antidepressant mechanisms of ECS. However, there is a possibility that ECS also increased other myelinated axons, including other subtypes of interneurons.

Some limitations of this study should be acknowledged. First, we conducted an ex vivo VBM analyses, but not an in vivo VBM assessment, in this study to detect the hippocampal volume change. Compared to in vivo VBM analyses, the fixation procedure consisting of paraformaldehyde (PFA) and phosphate buffer solution (PBS) strongly influences volume changes [45]. The volume changes depend on the duration, for which the brain was immersed in PFA for post-fixation and in PBS for storage. In this study, the duration of PFA and PBS immersion was strictly controlled for all samples to minimize the duration-dependent differences in ex vivo brain volume. Ex vivo VBM analysis can obtain high quality data with high spatial resolution, high signal-noise ratio, and high contrast, as compared to in vivo. Second, this study was conducted using wild-type mice. Our research questions in this study were to investigate if ECS would induce MRI-detectable hippocampal volume changes, and whether neurogenesis was necessary for this change. Because we confirmed in this study that ECS induced MRI-detectable brain volume increases in mice that were similar to human studies, a future study should include a mouse model of depression to explore how the MRI / histological changes might be associated with the potent antidepressant-like effect of ECS. Furthermore, the developed experimental and analysis pipeline can be used to probe the cellular mechanisms of other treatment interventions and may provide microstructural evidence for results from human MRI studies. Third, although we observed ECS-induced hippocampal volume increase in mice lacking neurogenesis, there is a possibility that neurogenesis could have an additive effect on the brain volume change following ECS. Fourth, the use of thinner slices (e.g., 40μm) in the Golgi staining means that the measured dendrite length might not accurately represent the true length, as an entire single neuron may not be fully visualized.

### Supplementary information

Revised supplementary information(uploaded version)

## Data Availability

All data in this study are available from the corresponding author upon request.
