# Supplementary material for: Neurogenesis-independent mechanisms of MRI-detectable hippocampal volume increase following electroconvulsive stimulation
Source: Neuropsychopharmacology. 2024 Jan 9;49(8):1236–45. doi: 10.1038/s41386-023-01791-1 (PMC11224397; doi:10.1038/s41386-023-01791-1)
Supplement: Supplementary file 1 — Revised supplementary information(uploaded version) [file 41386_2023_1791_MOESM1_ESM.docx]

**Supplemental Files**

**Neurogenesis-independent mechanisms of MRI-detectable hippocampal volume increase following electroconvulsive stimulation**

Yoshifumi Abe, Kiichi Yokoyama, Tomonobu Kato, Sho Yagishita, Kenji F Tanaka, Akihiro Takamiya

**Supplemental Materials and Methods**

**ECS**

Following anesthesia via 3.0% sevoflurane, ECS was administered to mice once daily, three times a week to simulate the ECT procedure in humans. To investigate the effect of the number of ECS sessions on brain volumetric changes, mice were allocated to four groups based on the number of ECS sessions (i.e., 0, 3, 6, and 9 sessions). ECS was given via bilateral ear clip electrodes with 25 mA and 0.5 msec pulse width for 1 second at a frequency of 100 Hz square wave pulses (UgoBasile, Comerio, Italy). Although different parameters have been explored in previous animal studies [33,34,46-48], we selected these parameters as seizures were consistently induced in our laboratory setting (Fig. 1B). The sham-treated group was exposed to the same procedure on the same schedule without electrical stimulation.

**X-ray irradiation**

Mice were deeply anesthetized by a mixture of medetomidine (0.75 mg/kg, i.p, Nipon Zenyaku Kogyo Co., Ltd., Hukushima, Japan), midazolam (4 mg/kg, i.p., Sandoz, Tokyo, Japan), and butorphanol (5 mg/kg, i.p., Meiji Seika Pharma, Co, Ltd., Tokyo, Japan). The heads of the mice were fixed in a small customized stereotaxic apparatus equipped with a lead plate with a 4-mm slit, which could be positioned freely (Narishige Scientific Instrument, Tokyo, Japan). The lead plate was placed above the mice to protect their head and body from radiation exposure to minimize the effect of the radiation on other brain regions. The slit in the lead plate was placed just above the whole hippocampus so that other brain regions were not exposed to the radiation. The head-fixed mice protected by the lead plate, were placed in the X-ray irradiator (AB-160, AcroBio Corporation, Tokyo, Japan). A total dose of 10 Gy was administered to the hippocampus.

In the X-ray + 9×ECS group, mice underwent ECS nine times using the protocol mentioned earlier, one week after irradiation. The CTL group received the same anesthesia used for the ECS three times a week over three weeks. The X-ray group received the same anesthesia used for ECS three times a week over three weeks, at one week after irradiation.

**EEG recording**

Surgeries were performed using a stereotaxic apparatus (SM-6M-HT, Narishige Scientific Instrument, Tokyo, Japan). Mice were deeply anesthetized with ketamine (100 mg/kg, i.p.) and xylazine (10 mg/kg, i.p.). The mice received permanent EEG electrode implants. Using a carbide cutter (drill diameter: 0.8 mm), five pits were drilled into the skull while avoiding penetration to prevent brain damage. Each implant had a 1.0-mm diameter stainless steel screw, which served as an EEG electrode. Two implants were placed over the frontal cortical area bilaterally (+1.0 mm anteroposterior and ±1.5 mm mediolateral from bregma) as reference electrodes, and two others over the parietal area bilaterally (+1.0 mm anteroposterior and ±1.5 mm mediolateral from lambda) as signal electrodes. Another electrode was placed over the right cerebellar cortex (−1.0 mm anteroposterior and +1.5 mm mediolateral from bregma) as the ground electrode. Finally, the electrode assembly was fixed to the skull using SuperBond (Sun Medical Co., Shiga, Japan).

The EEG signals were amplified (gain ×1000) and filtered (1-300 Hz) using a DC/AC differential amplifier (AM-3000, AM systems). The input was then received via an input module (NI-9215, National Instruments), digitized at a sampling rate of 1000 Hz using a data-acquisition module (cDAQ-9171, National Instruments), and recorded using a custom-made LabVIEW program (National Instruments). While the ECS current was being delivered, the EEG recording was stopped and then immediately restarted after the current delivery ended.

**MRI**

An *ex vivo* MRI was performed using a 11.7 T BioSpec 117/11 US/R unit (Biospin GmbH, Ettlingen, Germany) and a volume-type coil with a 23-mm inner diameter for transmitting and receiving. One day after the last ECS session, mice were deeply anesthetized with ketamine (100 mg/kg) and xylazine (10 mg/kg) and perfused with a 4% paraformaldehyde phosphate buffer solution. The brains were removed along with the skull and postfixed in the same fixative for 24 hours. The fixed brains were then stored in PBS for one week. The duration of PFA and PBS immersion was consistent for all samples to avoid differences in brain volume caused by postperfusion immersion fixation and storage[45]. Brains within their skulls were firmly fixed using fitted sponges into an acrylic tube (22-mm diameter) filled with Fluorinert (Sumitomo 3M Limited, Tokyo, Japan) to minimize the signal intensity attributed to the embedding medium. Additionally, vacuum degassing was performed to reduce air bubble-derived artifacts on the structural images. For brain volumetric analysis, the structural images were acquired using T2-weighted multi-slice rapid acquisition with relaxation enhancement (RARE) with the following parameters: repetition time = 2000 ms, echo time = 75 ms, spatial resolution = 100 × 100 × 100 µm, RARE factor = 32, and averages=8.

Image preprocessing and statistics for the whole-brain voxel-based analysis were performed using SPM12 (Wellcome Trust Centre for Neuroimaging, London, UK), and in-house software written in MATLAB (MathWorks, Natick, MA, USA). First, each T2-weighted image was resized by a factor of 10 to account for the whole-brain volume differences between humans and rodents, and aligned to the same space by registering each image to tissue probability maps (TPMs). Next, each image was segmented into gray matter, white matter, and cerebrospinal fluid using a unified segmentation approach, which enables image registration, tissue classification, and bias correction. The segmented images were spatially normalized into the study-specific template in dose-dependent and X-ray irradiation experiments, respectively, which was created through a Diffeomorphic Anatomical Registration Through Exponentiated Lie Algebra (DARTEL) algorithm. Modulated gray matter images were then obtained for each animal by the determinant of the Jacobian of the transformation to account for the expansion and/or contraction of the brain regions. These images were smoothed with a 3-mm full width at half maximum Gaussian kernel. The total brain volume (TBV) was defined as a sum of the GMV and the white matter volume (WMV).

Whole-brain voxel-wise comparisons of GMV between the groups were performed using SPM12 with the TBV as a covariate. The first analysis compared the GMV between the ECS-treated mice (ECS) and sham-treated mice (CTL). A whole-brain regression analysis, including the number of ECS sessions (i.e., three, six, and nine) as an independent variable and the TBV as a covariate of no interest, was also conducted to identify the brain regions which correlated with the number of ECS sessions. A second analysis compared the GMV between the X-ray-irradiated mice treated with ECS (X-ray+ECS) and the X-ray-irradiated mice treated with sham ECS (X-ray). The significance threshold for the voxel-wise whole-brain analyses was set at a cluster-level family-wise error (FWE) corrected p <0.05, with an individual voxel threshold of p = 0.001.

To identify the dorsal and ventral hippocampus, we utilized the pattern of *Wfs1* gene expression found at the website of the Allen Institute for Brain Science (AIBS) for gene expression (Experimental ID: 74881161). The energy intensity of the *Wfs1* gene expression was registered to the study-specific mouse T2 template using Advanced Normalization Tools (ANTs) (https://github.com/ANTsX/ANTs). To identify the hippocampal subregions including the DG, CA3, CA2, and CA1, we utilized a mouse brain annotation atlas which we previously developed, based on the Allen Brain Atlas [28]. The atlas was also registered to the study-specific mouse T2 template using ANTs.

An ROI-based analysis was performed using the mouse brain annotation atlas. We defined ROIs including the whole dorsal, and ventral hippocampus, and the hippocampal sub-regions. The ROIs of the DG, CA3, CA2, and CA1 were combined, and Voxel values (Jacobian modulation values) of each ROI were extracted using MarsBaR (https://marsbar-toolbox.github.io/index.html). The brain volume of each defined region was estimated by multiplying the average voxel values on the ROI by the number of voxels in the ROI and the size of one voxel on the T2 image (100 × 100 × 100 µm).

**Histology**

Perfusion fixation was performed the day after the last ECS. Mice were deeply anesthetized with ketamine (100 mg/kg, i.p.) and xylazine (10 mg/kg, i.p.) and perfused with a 4% paraformaldehyde phosphate buffer solution. The brains were removed from the skull and postfixed in the same fixative overnight. Subsequently, the brains were cryoprotected in 20% sucrose overnight and frozen. The frozen brains were cut on a cryostat at a 25-µm thickness for the *in situ* hybridization (ISH) and mounted on silane-coated glass slides (Matsunami Glass, Osaka, Japan). The floating brain sections for immunostaining were cut on a cryostat at a 40-µm thickness. The sections were incubated with the primary antibodies overnight at room temperature. The following antibodies were used: anti-VGAT (1:1000 dilution; guinea pig polyclonal, Frontier Institute, Hokkaido, Japan); anti-PV (1:1000 dilution; goat polyclonal, Frontier Institute); anti-DCX (1:250 dilution; mouse monoclonal, clone E-6, sc-271390); anti-PLP (1:1 dilution; rat monoclonal, clone AA3 hybridoma supernatant); anti-green fluorescent protein (GFP) (1:250 dilution, goat polyclonal; Rockland Immunochemicals, Pottstown, PA, USA), anti-NeuN (1:1000 dilution, rabbit monoclonal, EPR12763, Abcam, Cambridge, UK); anti-regulator of G protein signaling 14 (RGS14) (1:1000 dilution, mouse monoclonal, N133/21, NeuroMab, CA, USA); anti-PSD95 (1:1000 dilution; goat polyclonal, Frontier Institute); and anti-VGluT1 (1:1000 dilution; rabbit polyclonal, Frontier Institute). The sections were then treated with species-specific secondary antibodies conjugated to Alexa Fluor 488, 555, 594, or 647 for 2 hours at room temperature. Mounting medium (ProLong Glass Antifade Mountain, Thermo Fisher Scientific, Waltham, MA, USA) was applied to the samples and they were mounted with coverslips (thickness: No.1, Matsunami Glass). Macro-fluorescent images were obtained with an inverted microscope (BZ-X710; Keyence, Osaka, Japan) or a confocal microscope (LSM710; Carl Zeiss, Oberkochen, Germany). Micro-fluorescent images were obtained using super-resolution microscopy (SRM).

**Super-resolution microscopy (SRM)**

Structured illumination microscopy (SIM)-SRM images were obtained using a Zeiss ELYRA 3D-SIM system equipped with an EM-CCD camera (Carl Zeiss). Before obtaining the SIM-SRM images, precise alignment for the different wavelengths of light was performed using the same mounting medium (ProLong Glass) containing 0.1% Tetraspeck (0.2-µm beads, Thermo Fisher Scientific) to correct for the unavoidable laser misalignment and optical aberrations, which can lead to alignment errors at very high resolutions. Next, 14–20 Z-section images were obtained at intervals of 130 nm using a 64× objective lens. The number of pattern rotations for the structured illumination was adjusted to three in the ELYRA system. After obtaining all images, the SIM images were reconstructed and aligned using the channel alignment data.

**Analysis of the SRM images**

The SRM images were analyzed using ImageJ (http://rsb.info.nih.gov/ij/). Optimal brightness and grayscale pixel values were manually adjusted to provide the sharpest discrimination of the microstructure border. These adjusted images were then converted into binary images. To calculate the density of the VGAT^+^, VGluT1^+^, or PSD95^+^ puncta, SRM images for each stain were cut to 80 μm×80 μm in size and obtained at three random locations in each hippocampal layer (SO, SR, and SLM of vCA1). The number of puncta were counted and the mean of the three locations was calculated. To calculate the puncta area, 3D-SRM images for each stain were randomly obtained at three locations each within the hippocampal layers. For the area calculation, we looked at the 3D image and used the single plane in which the area was the largest. We measured the area of 150 puncta from each of the three locations. The mean area of the 150 puncta was taken as the representative puncta area for each animal. To calculate the area of the NeuN^+^ soma of the vCA1 neurons, 3D-SRM images of NeuN staining were randomly obtained. We looked at the 3D image and used the single plane in which the area was the largest for the calculation. We calculated the area of 50 somas per mouse and took the mean as the representative soma area for each animal. The methods used to measure the axon diameter, myelin thickness, and g-ratio (the ratio of the inner axonal diameter to the total outer diameter) have been previously described[49,50]. SRM images of the PLP stain were cut to 80 μm×80 μm in size and obtained at three random locations in each hippocampal layer. The axonal diameter was defined as the minor axis of an ellipse-approximated axon. The median axonal diameter over 50 myelinated axons was considered the representative value for each animal. The g-ratio was calculated using the equation 0.5AD / (0.5AD + MT), where AD is the axonal diameter and MT is the myelin thickness. The mean myelin thickness and g-ratio over 50 myelinated axons were considered representative for each animal. To calculate the percent area of PLP^+^ myelin, GLT1^+^ astrocytes and Iba1^+^ microglia, SRM images of PLP, GLT1, or Iba1 stains were cut to 80 μm×80 μm in size and randomly obtained at three locations within each of the hippocampal layers. The percent area was calculated using the equation: glial area / image area × 100, where grail area is the PLP^+^, GLT1^+^ or Iba1^+^ area and the image area is the area of the region. The average area at three locations was taken as the representative percent area of the myelin, astrocytes, or microglia for each animal.

**Measurement of thickness of the hippocampal layers**

Macro-fluorescent images of VGLuT1 staining were obtained with an inverted microscope (BZ-X710; Keyence, Osaka, Japan). The hippocampal layers were defined by the contrast of the VGluT1 staining. The thicknesses of the hippocampal layers were measured along the dendritic direction of the vCA1 neurons delineated by Golgi staining.

***in situ* hybridization**

The detailed protocol has been described previously[49]. The 25-µm sections were treated with 40 μg/mL proteinase K (Roche) for 30 minutes before being washed with PBS for 5 minutes and postfixed with 4% paraformaldehyde in PBS for 15 minutes to deactivate the proteinase K. After a 5-minute wash with PBS, the sections were acetylated with 0.25% acetic anhydride. Prehybridization was conducted for 2 hours at 60°C in prehybridization buffer containing 50% formamide (Wako, Tokyo, Japan), 50× Denhardt’s solution (Nacalai Tesque, Kyoto, Japan), and 10 mg/mL salmon sperm DNA (Invitrogen, Tokyo, Japan). After removing the prehybridization buffer, sections were hybridized overnight at 60°C in hybridization buffer containing the following digoxigenin-labeled cRNA probes: colony-stimulating factor 1 receptor (*Csf1r*, a marker of microglia), *Plp1* (an oligodendrocyte marker), and *Gja1* (connexin 42, an astrocyte marker). After the sections were washed in buffers with serial differences in stringency, they were incubated with an alkaline phosphatase-conjugated anti-digoxigenin antibody (1:5000; Roche) for 90 minutes at room temperature. Unbound antibody was removed by four 10-minute washes. The cRNA probes were visualized by being incubated with a freshly prepared colorimetric substrate (NBT/BCIP; Roche) overnight at room temperature. Following the ISH staining, the sections were counterstained with nuclear fast red (Sigma-Aldrich) and images were captured using an inverted light microscope (BZ-X710, Keyence).

**Cell counts on the ISH images**

To evaluate the cell density of astrocytes, microglia, and oligodendrocytes, cell counts were performed from the ISH images using ImageJ. In one slice, the SR layers of the vCA1 were identified by contrast with the nuclear fast red staining and in reference to the Paxinos and Franklin mouse brain atlas. We defined an ROI of the size 0.2 mm×0.4 mm in the SR layer (See Supplemental Fig. 10). The cell density in the SR layer was calculated by dividing the number of cells expressing the marker RNA in the ROI by the area of the ROI.

**Golgi staining and analysis**

Mice were deeply anesthetized with ketamine (100 mg/kg, i.p.) and xylazine (10 mg/kg, i.p.) and perfused with a formaldehyde/glutaraldehyde fixative (BioEnno, Irvine CA). The brains were removed from the skull and postfixed in the same fixative overnight. Brains were sliced to 60 μm on a vibratome (Leica) and floating sections were collected in PBS. On-slice Golgi staining was performed using a SliceGolgi kit (BioEnno, Irvine CA). Staining was performed according to the kit protocol, using a 7-day Golgi impregnation. Slices were mounted on glass slides and allowed to dry, then dehydrated in 100% EtOH, and cleared with xylenes. Slides were coverslipped using Permount. Images were captured using an inverted light microscope (BZ-X710, Keyence) with a 10× objective lens for analyzing dendritic arborization and with a 100× objective lens for analyzing the spine density and size.

The Golgi stained images were analyzed using ImageJ. Analysis of the dendritic arborization (Sholl analysis) was performed using an ImageJ plugin program, simple neurite tracer (SNT)[51]. Neurites were traced following their position along the z-axis for optimal accuracy. Reconstruction of the somata and dendrites was performed using the ROI manager tool. First and subsequent shells of the Sholl analysis were set to a radius of 20 μm and the intersections at each Sholl radius were determined. For analysis of the spine density and size, the spines were counted along the primary dendrite of the large apical and the basal dendrites in single vCA1 neurons. Only cells with clearly visible dendrites and easily identifiable structures and soma were counted. The spines were counted in the SR layer. To allow for the data comparison and determination of relative spine density, the total number of spines was standardized by the length of the dendrites. The spine head diameter was counted at the primary dendrite of the large apical dendrite and the basal dendrite of the vCA1 neurons, and was defined as the minor axis of an ellipse-approximated spine head. More than 10 neurons were measured in each animal and the mean spine density and mean spine head diameter were considered representative values for each animal.

**RNAseq**

Sample collection was performed the day after the last ECS. CTL and 9×ECS mice were deeply anesthetized with ketamine (100 mg/kg, i.p.) and xylazine (10 mg/kg, i.p.) and their brains were quickly removed. A brain slice (approximately −3.0 mm form the bregma) containing the ventral hippocampus was sectioned to a thickness of 1 mm, and the ventral hippocampal tissue was subsequently dissected.

The total RNA was isolated using TRIzol Reagent (#15596018, Thermo Fisher Scientific, Waltham, MA, USA), and purified using an RNeasy Micro Kit (74004, Qiagen) according to the manufacturer’s instructions, which included a step involving incubation with DNase. Purified RNA was quantified using a NanoDrop 2000 (Thermo Scientific) and Agilent Technologies Bioanalyzer 2100 RNA Pico chips (5067-1513, Agilent Technologies), according to the manufacturer’s instructions. The RNA integrity number (RIN) in all cases was > 7. The total RNAs were subsequently processed to generate an mRNA-seq library using a NEBNext Poly(A) mRNA Magnetic Isolation Module (NEB, E7490S), and NEBNext Ultra II Directional RNA Library Prep with Sample Purification Beads (NEB, E7765S) according to the manufacturer’s protocol. The libraries were sequenced for a 150-bp paired-end read by an Illumina NovaSeq 6000. For each sample, the quality control of the reads was inspected using the FastQC tool. Low-quality reads and adapter sequences were removed using Trimmomatic[52]. RNA sequencing reads were aligned to the mouse reference genome using HISAT2[53]. After alignment, the transcripts were assembled and counted by feature-Counts[54]. Expression level estimation was reported as a transcripts per million (TPM) value.

The differential expression analysis to compare between CTL and ECS groups was performed using in-house software written in MATLAB. Resulting P values were adjusted using the Benjamini and Hochberg approach to control for the false-discovery rate (FDR). Differentially expressed genes (DEGs) identified with a threshold p value < 0.05 and fold change ≥ 1.5 were selected for further analysis. Gene ontology (GO) analysis was carried out using The Database for Annotation, Visualization and Integrated Discovery (DAVID)[55] to identify the functional enrichment, which was categorized by biological process (BP) and cellular component (CC). The upregulated and downregulated DEGs were separately analyzed by the DAVID database (http://david.ncifcrf.gov/summary.jsp). The DAVID GO terms featuring an FDR-corrected p value of < 0.05 were considered significantly enriched.

**Statistical analysis**

Statistical processing was performed using R, MATLAB and Excel (Microsoft, Redmond, WA, USA) software. In the histological studies and ROI-based analyses, both a two-tailed Student’s t-test and an analysis of variance (ANOVA) were employed. Bonferroni corrections were applied to correct for multiple comparisons. Pearson’s correlation coefficients were calculated to investigate the relationship between MRI-derived values and histological data. In this analysis, normalized voxel values were extracted from clusters identified in the whole-brain VBM analysis. Values are shown as a mean and standard error of the mean (SEM), and are plotted as scatter diagrams.

**Supplemental references**

46 Kaastrup Muller H, Orlowski D, Reidies Bjarkam C, Wegener G, Elfving B. Potential roles for Homer1 and Spinophilin in the preventive effect of electroconvulsive seizures on stress-induced CA3c dendritic retraction in the hippocampus. Eur Neuropsychopharmacol. 2015;25(8):1324-31.

47 Maynard KR, Hobbs JW, Rajpurohit SK, Martinowich K. Electroconvulsive seizures influence dendritic spine morphology and BDNF expression in a neuroendocrine model of depression. Brain Stimul. 2018;11(4):856-59.

48 Chen F, Danladi J, Wegener G, Madsen TM, Nyengaard JR. Sustained Ultrastructural Changes in Rat Hippocampal Formation After Repeated Electroconvulsive Seizures. Int J Neuropsychopharmacol. 2020;23(7):446-58.

49 Zhang TR, Guilherme E, Kesici A, Ash AM, Vila-Rodriguez F, Snyder JS. Electroconvulsive Shock, but Not Transcranial Magnetic Stimulation, Transiently Elevates Cell Proliferation in the Adult Mouse Hippocampus. Cells. 2021;10(8).

50 Jonckheere J, Deloulme JC, Dall'Igna G, Chauliac N, Pelluet A, Nguon AS, et al. Short- and long-term efficacy of electroconvulsive stimulation in animal models of depression: The essential role of neuronal survival. Brain Stimul. 2018;11(6):1336-47.

51 de Guzman AE, Wong MD, Gleave JA, Nieman BJ. Variations in post-perfusion immersion fixation and storage alter MRI measurements of mouse brain morphometry. Neuroimage. 2016;142:687-95.

52 Takata N, Sato N, Komaki Y, Okano H, Tanaka KF. Flexible annotation atlas of the mouse brain: combining and dividing brain structures of the Allen Brain Atlas while maintaining anatomical hierarchy. Sci Rep. 2021;11(1):6234.

53 Abe Y, Komaki Y, Seki F, Shibata S, Okano H, Tanaka KF. Correlative study using structural MRI and super-resolution microscopy to detect structural alterations induced by long-term optogenetic stimulation of striatal medium spiny neurons. Neurochem Int. 2019;125:163-74.

54 Yamazaki Y, Abe Y, Fujii S, Tanaka KF. Oligodendrocytic Na(+)-K(+)-Cl(-) co-transporter 1 activity facilitates axonal conduction and restores plasticity in the adult mouse brain. Nat Commun. 2021;12(1):5146.

55 Arshadi C, Gunther U, Eddison M, Harrington KIS, Ferreira TA. SNT: a unifying toolbox for quantification of neuronal anatomy. Nat Methods. 2021;18(4):374-77.

56 Bolger AM, Lohse M, Usadel B. Trimmomatic: a flexible trimmer for Illumina sequence data. Bioinformatics. 2014;30(15):2114-20.

57 Kim D, Langmead B, Salzberg SL. HISAT: a fast spliced aligner with low memory requirements. Nat Methods. 2015;12(4):357-60.

58 Liao Y, Smyth GK, Shi W. featureCounts: an efficient general purpose program for assigning sequence reads to genomic features. Bioinformatics. 2013;30(7):923-30.

59 Huang da W, Sherman BT, Lempicki RA. Systematic and integrative analysis of large gene lists using DAVID bioinformatics resources. Nat Protoc. 2009;4(1):44-57.

**Supplemental figures and legends**


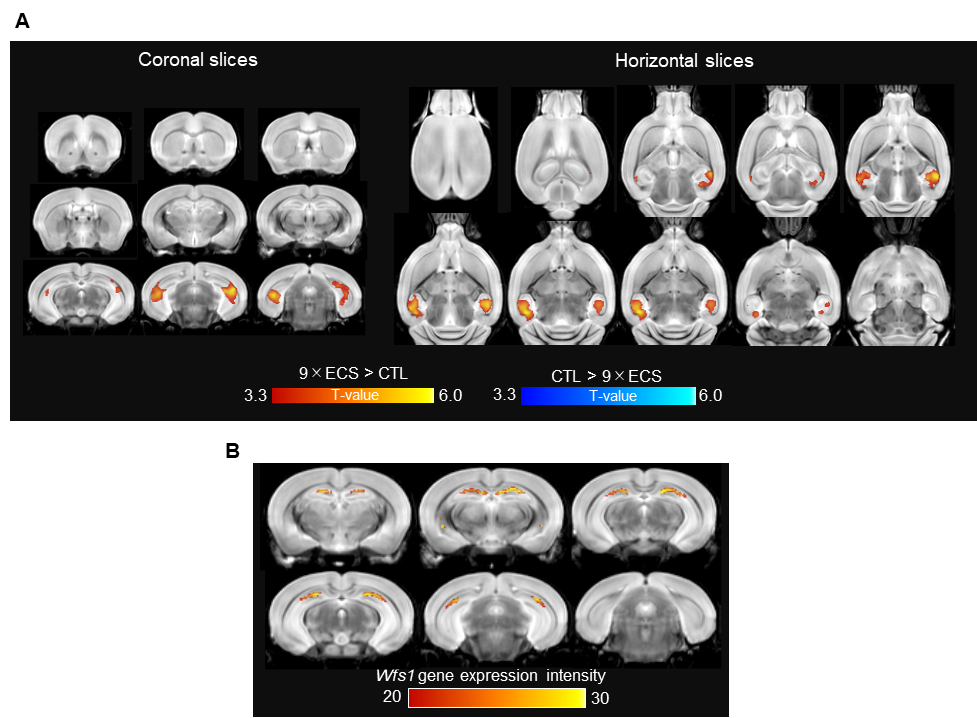


**Supplemental Fig. 1. ECS-induced volume increases are observed mainly in the ventral hippocampus.**

**(A)** Results of the whole brain voxel-wise analysis on coronal and horizontal brain slices. The red color represents volume increases, while the blue color represents volume decreases. The results are shown at a liberal statistical threshold (i.e., uncorrected p <0.001). There were no brain regions that showed volume decreases at a predefined cluster-level FWE-corrected p <0.05. **(B)** The *Wfs1* gene expression intensity was overlaid on the T2 brain image. We defined the regions showing higher expression of *Wfs1* (intensity>20) as the dorsal hippocampus and the regions showing the lower expression as the ventral hippocampus.


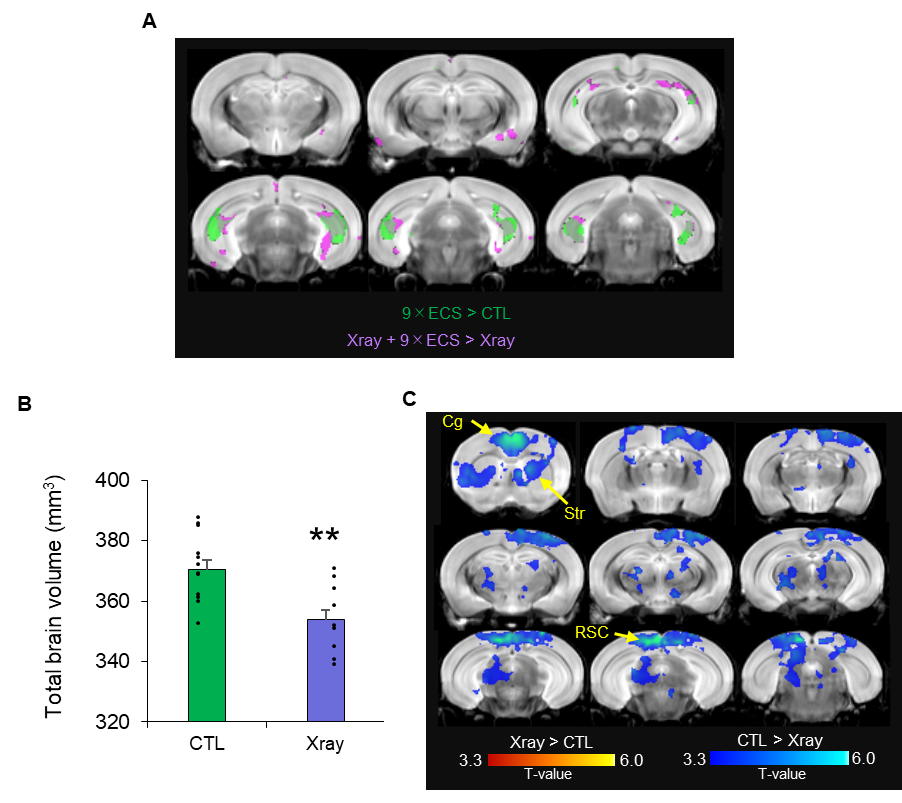


**Supplementary Fig. 2. X-ray irradiation decreases total brain volume.**

**(A)** Whole brain voxel-wise group comparison superimposed in green (9×ECS>CTL) and purple (Xray+9×ECS>Xray) which is shown in Fig. 2G and H. **(B)** The total brain volume (TBV) was compared between CTL and X-ray groups. **p<0.01 (Student’s t-test). **(C)** A whole brain voxel-wise group comparison between CTL (n=9) and X-ray (n=9). The TBV was not applied as a covariate of no interest to explore brain regions contributing to the TBV reductions after X-ray irradiation. The blue color represents the volume decrease while the red color represents the volume increase. Cg; cingulate cortex, Str; striatum, RSC; retrosplenial cortex.


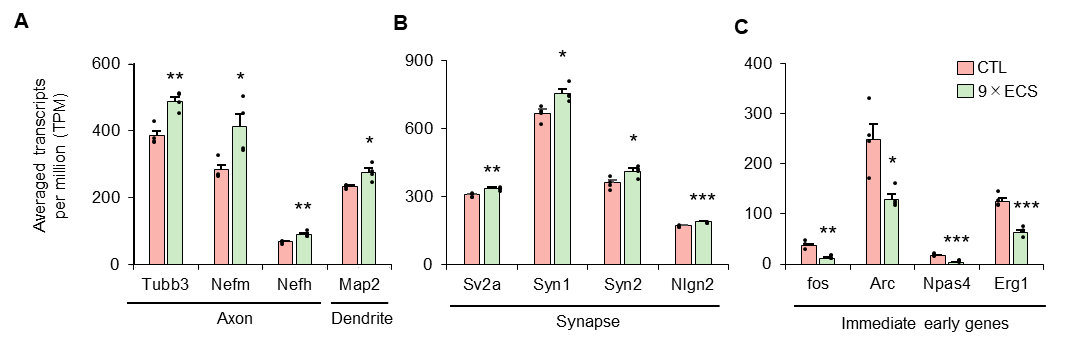


**Supplemental Fig. 3. ECS increases the expression level of microstructural-related genes and decreases neural activity-related genes.**

**(A-C)** The expression levels of genes related to axons and dendrites, synapses, and the immediate early genes were compared between CTL (n=3) and ECS (n=3). * p<0.05, ** p<0.01 (Student’s t-test, p-values were Bonferroni corrected, vs. CTL).


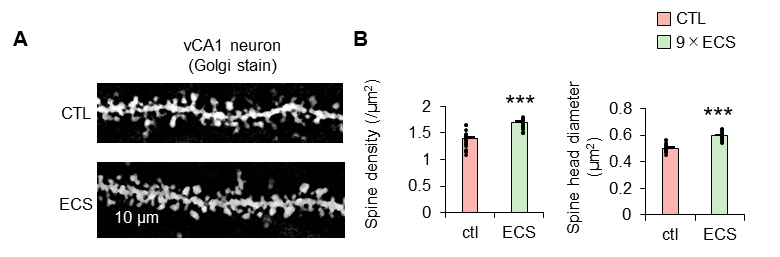


**Supplemental Fig. 4. ECS increases spine density and size of the vCA1 pyramidal neurons.**

**(A)** Representative Golgi stained images in the SR of the vCA1 neurons. **(B)** Spine density and spine head diameter were compared in the SR of the vCA1 between CTL and ECS (20 neurons were counted from three mice of each group). *** p<0.001 (Student’s t-test, vs. CTL).


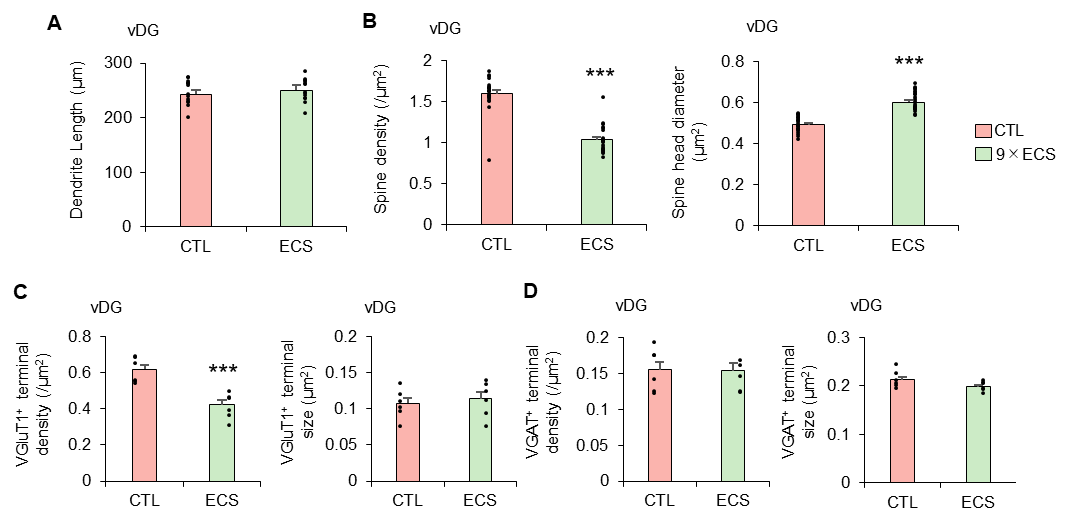


**Supplementary Fig. 5. ECS reduces the spine density in vDG neurons.**

**(A)** A comparison of DG dendrite length in Golgi-stained samples between CTL and 9×ECS groups (15 neurons from three mice per group were analyzed). **(B)** A comparison of spine density and spine head diameter in Golgi-stained vDG neurons between CTL and 9×ECS groups (20 neurons from three mice per group were analyzed). **(C)** The density and size of the VGluT1+ excitatory terminals were compared in the vDG between CTL (n=5) and 9×ECS (n=5) groups. **(D)** The density and size of the VGAT+ inhibitory terminals were compared in the vDG between CTL (n=5) and 9×ECS (n=5) groups. ***p<0.001 (Student’s t-test, vs. CTL).


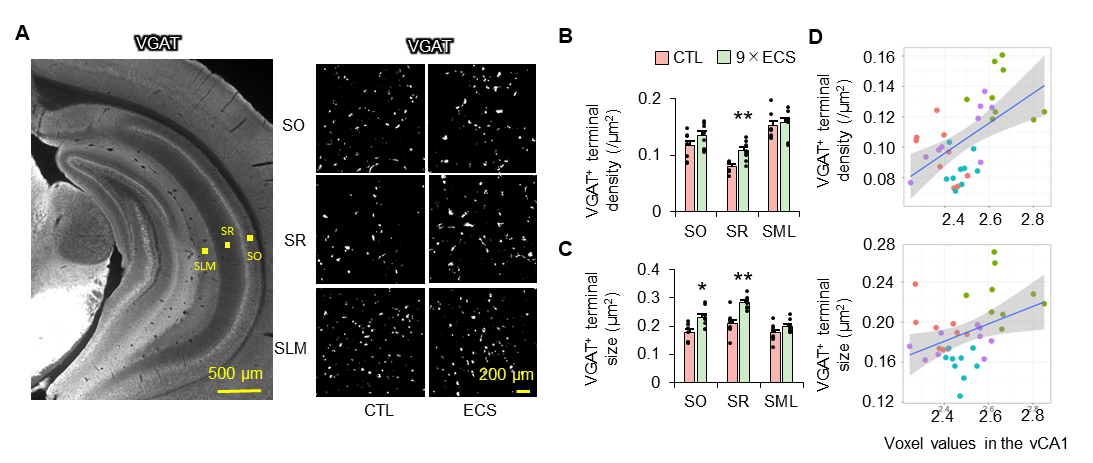


**Supplemental Fig. 6. ECS induces microstructural changes in inhibitory synapses.**

**(A)** A representative fluorescence microscope image of VGAT staining (left) and SRM images (right) in each layer of the vCA1. **(B, C)** The density and size of the VGAT^+^ inhibitory terminals in each layer were compared between CTL (n=8) and ECS (n=8). A two-way repeated ANOVA was performed (Density: ECS p=0.0075, interaction p=0.31, Size: ECS p=2.5×10^-6^, interaction p=0.075). **(D)** Scatter plots of the density or size of the VGAT^+^ inhibitory terminals in the SR and normalized voxel values in the vCA1. There was a significant correlation of the voxel values in the vCA1 with both the VGAT+ terminal density (r=0.55, df=34, p<0.001, R2=0.30) and VGAT+ terminal size (r =0.39, df=34, p=0.02, R2=0.15). * p<0.05, ** p<0.01 (Student’s t-test, p-values were Bonferroni corrected, vs. CTL).


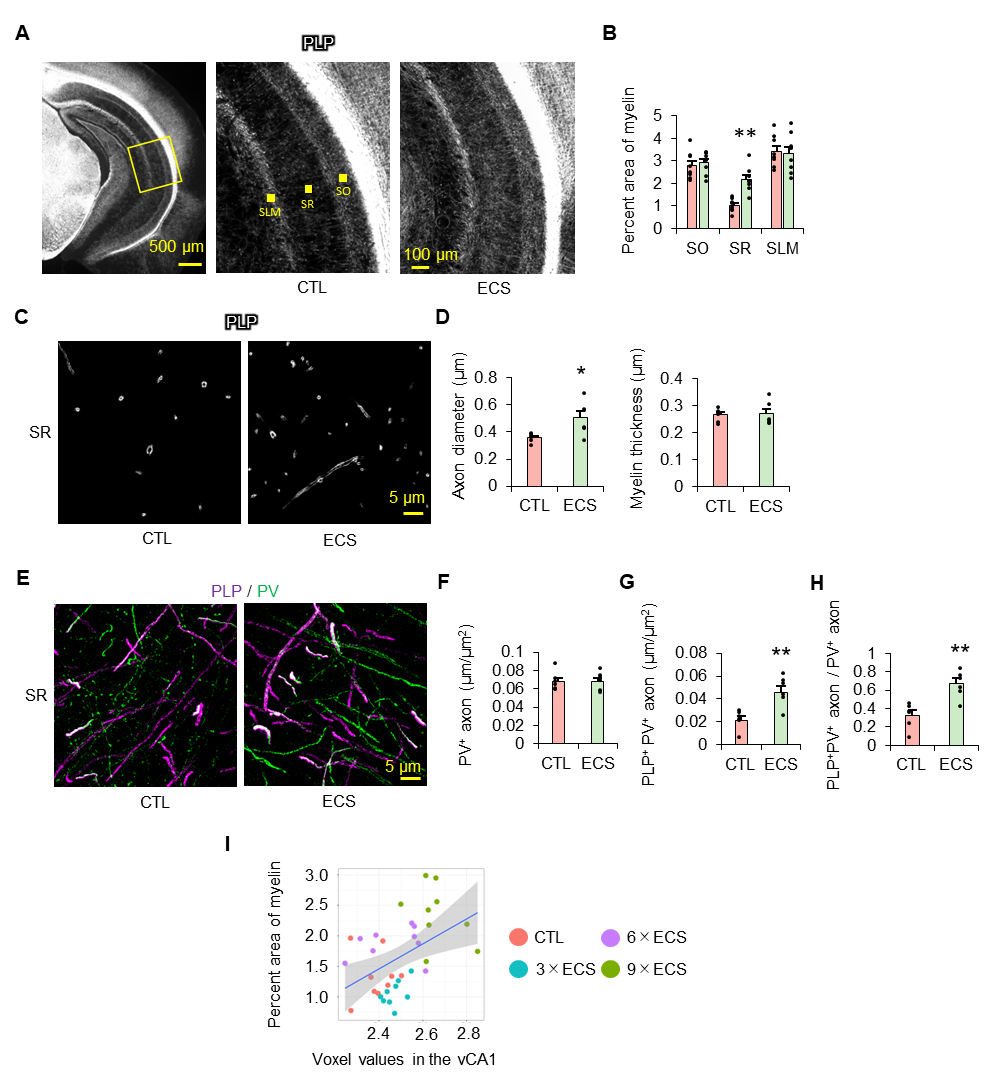


**Supplemental Fig. 7. ECS induces microstructural changes in myelin.**

**(A)** A representative fluorescence microscope image of the PLP staining. **(B)** The percent area of PLP^+^ myelin in each layer of the vCA1 was compared between CTL (n=8) and ECS (n=8). A two-way repeated ANOVA was performed (ECS p=0.034, interaction p=0.015). **(C)** Representative SRM images of PLP staining in the SR of the vCA1. **(D)** The axon diameter and myelin thickness of the axons were compared in the SR of the vCA1 between CTL (n=6) and ECS (n=6). **(E)** Representative SRM images of PLP and Parvalbumin (PV) staining in the SR of the vCA1. **(F-G)** The density of the PV^+^ axons by unit area, the density of the PLP and PV double positive axons by unit area, and the ratio of the PLP and PV double positive axons to the PV^+^ axons were compared in the SR of the vCA1 between CTL (n=6) and ECS (n=6). ECS increased myelin in the PV^+^ inhibitory interneurons in the vCA1. **(I)** Scatter plots of the percent area of PLP in the SR and the volume values in the vCA1. There was a significant correlation of the voxel values in the vCA1 with the percent area of PLP (r =0.47, df=34, p=0.004, R^2^=0.22). The voxel values were normalized using the total brain volume of each animal and then the values for the left and right vCA1 were averaged. * p<0.05, ** p<0.01 (Student’s t-test, p-values were Bonferroni corrected, vs. CTL).


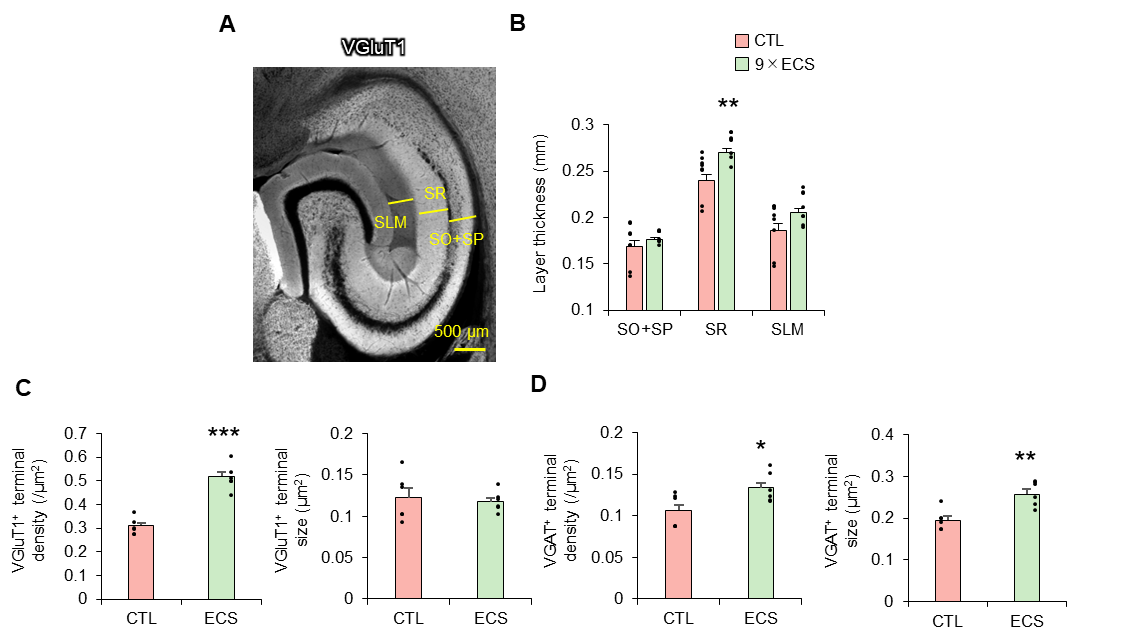


**Supplementary Fig. 8. The increases in the SR thickness and terminal density were confirmed by the same histological assessments on horizontal slices.**

**(A)** Representative VGluT1 staining of horizontal slices. SO: stratum oriens; SP: stratum pyramidale; SR: stratum radiatum, SLM: stratum lacunosum moleculare. **(B)** Each layer thickness in the vCA1 was compared between CTL (n=5) and 9×ECS (n=5). A two-way repeated ANOVA was performed (ECS p=0.00095, interaction p=0.23). **(C)** The density and size of the VGluT1+ excitatory terminals were compared in the vCA1 between CTL (n=5) and 9×ECS (n=5). **(D)** The density and size of the VGAT^+^ inhibitory terminals were compared in the vCA1 between CTL (n=5) and 9×ECS (n=5). *p<0.05, **p<0.01, ***p<0.001 (Student’s t-test, vs. CTL).


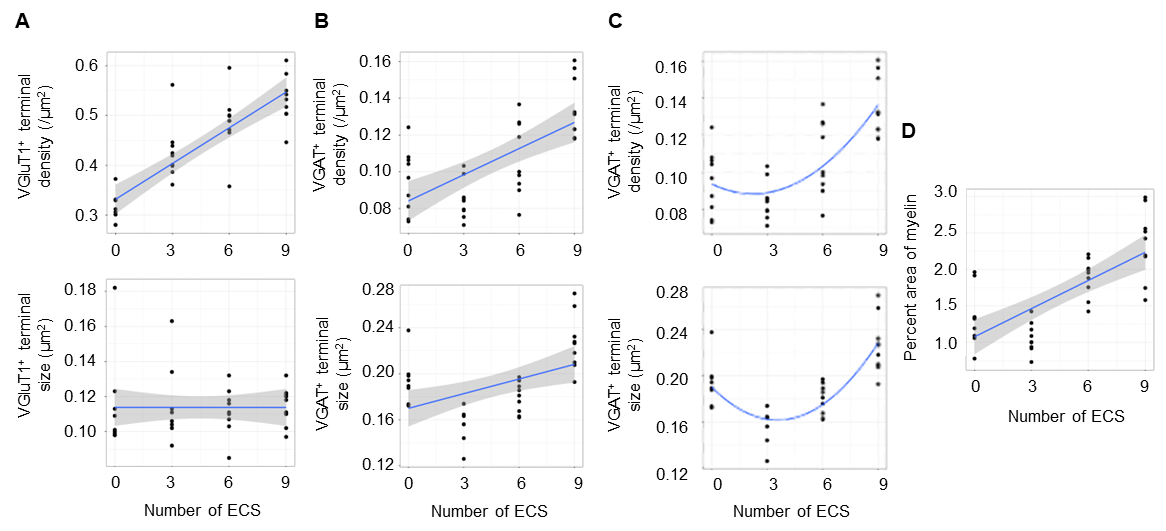


**Supplemental Fig. 9. Excitatory terminal density correlates significantly with MRI-defined hippocampal volume.**

**(A)** A linear correlation analysis between the density or size of the VGluT1^+^ excitatory terminals in the SR and the number of ECS sessions (n=9 for each session). **(B, C)** Linear and nonlinear correlations between the density of VGAT^+^ inhibitory terminals in the SR, or the size, respectively with the number of ECS sessions (n=9 for each session). Akaike Information Criteria (AIC) of the linear model for the relationship between the VGAT^+^ inhibitory terminal density and the number of ECS sessions was -178.9, whereas the nonlinear fitting model was -187.7. The AIC of the linear model for the relationship between the VGAT^+^ inhibitory terminal size and the number of ECS sessions was -151.5, whereas the nonlinear fitting model was -179.0. These results suggest that the nonlinear models fitted better than the linear models. **(D)** A linear correlation analysis between the percentage of the PLP^+^ myelin in the SR and the number of ECS sessions (n=9 for each session).


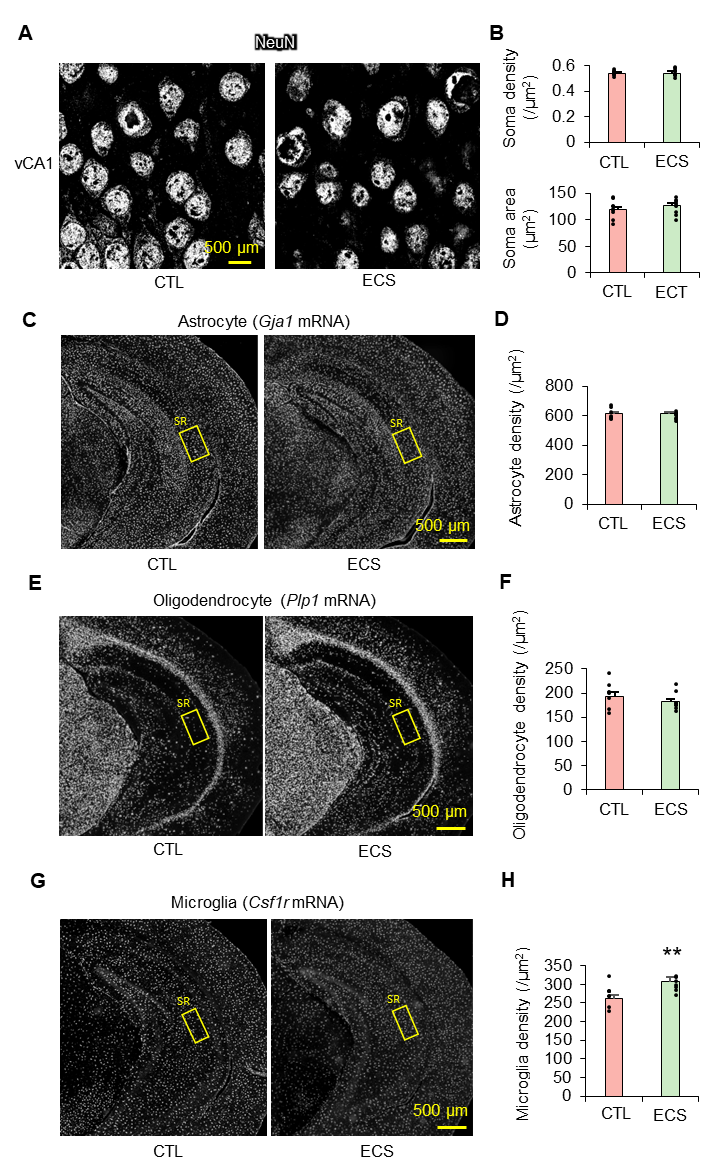


**Supplemental Fig. 10. ECS does not affect the number of neuronal and glial cells except for microglia.**

**(A)** Representative confocal microscopy images of NeuN staining in the SP of the vCA1. **(B)** The soma density and size of the NeuN^+^ vCA1 neurons were compared in the vCA1 between CTL (n=9) and ECS (n=9). **(C)** Representative ISH images of the *Gja1* mRNA in the vCA1. **(D)** The density of the *Gja1*^+^ astrocytes was compared in the SR of the vCA1 between CTL (n=8) and ECS (n=8). **(E)** Representative ISH images of the *Plp1* mRNA in the vCA1. **(D)** The density of the *plp1*^+^ oligodendrocytes was compared in the SR of the vCA1 between CTL (n=8) and ECS (n=8). **(C)** Representative ISH images of the *Csf1r* mRNA in the vCA1. **(D)** The density of the *Csf1r*^+^ microglia was compared in the SR of the vCA1 between CTL (n=8) and ECS (n=8). ** p<0.01 (Student’s t-test, vs. CTL).


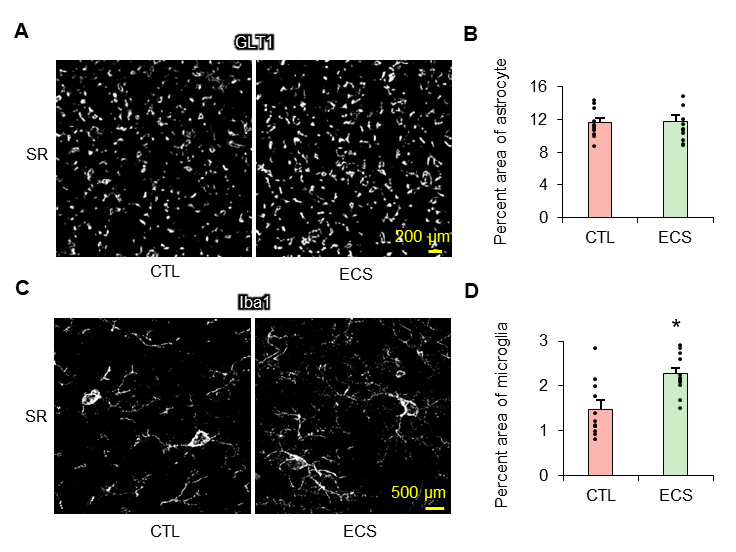


**Supplemental Fig. 11. ECS increases microglia volumes but does not affect astrocyte volumes.**

**(A)** Representative SRM images of GLT1 staining in the SR of the vCA1. **(B)** The percent area of GLT1^+^ astrocytes was compared in the vCA1 between CTL (n=12) and ECS (n=12). **(C)** Representative SRM images of Iba1 staining in the SR of the vCA1. **(D)** The percent area of the Iba1^+^ microglia was compared in the vCA1 between CTL (n=12) and ECS (n=12). * p<0.05 (Student’s t-test, vs. CTL).


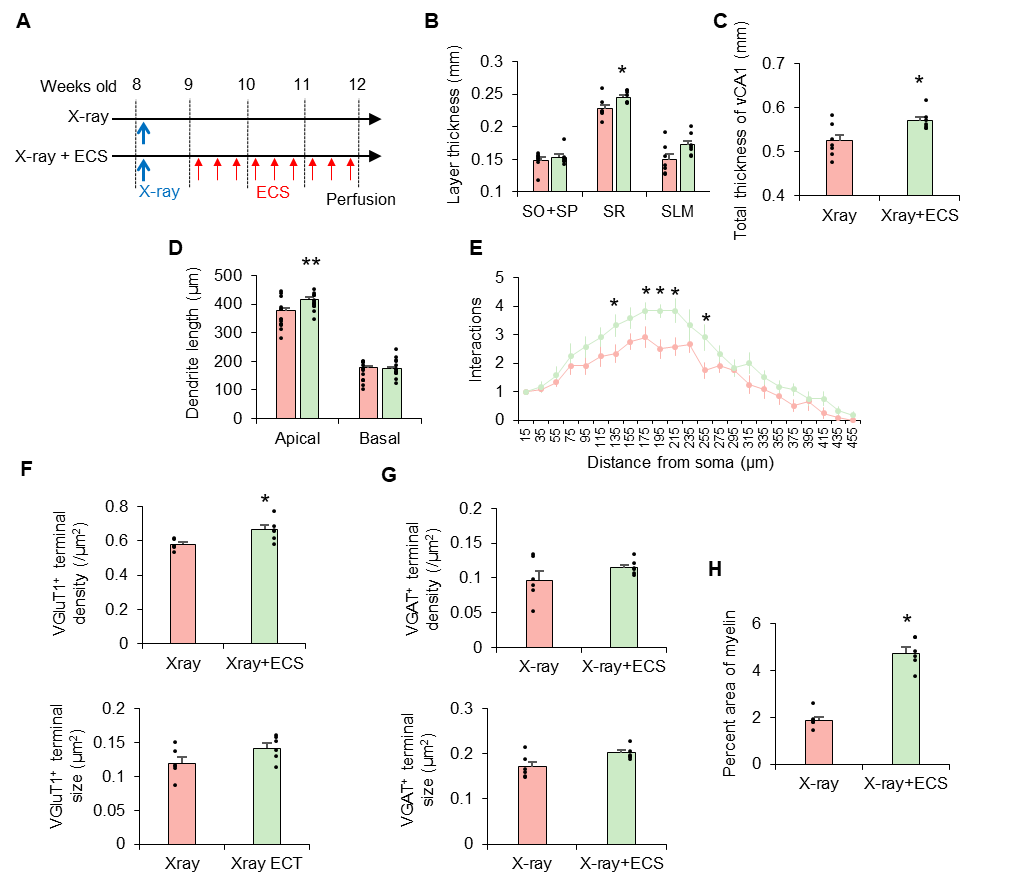


**Supplemental Fig. 12. ECS induces microstructural changes in the vCA1 even in mice lacking hippocampal neurogenesis.**

**(A)** The time course of ECS and X-ray irradiation. **(B, C)** Each layer thickness and total layer thickness (SO+SP+SR+SLM) in the vCA1 were compared between X-ray (n=7) and X-ray+ECS (n=7). A two-way repeated ANOVA was performed (Layer: ECS p=0.0031, interaction p=0.32). **(D)** The lengths of the apical or basal dendrites of the vCA1 neurons were compared between X-ray and X-ray+ECS (20 neurons were measured from three mice of each group). A two-way repeated ANOVA was performed (Dendrite: ECS p=0.0049, interaction p=0.12). **(E)** Interaction numbers were plotted in a Sholl analysis of the apical dendrites in the vCA1 neurons. A two-way repeated ANOVA was performed (ECS p=9.2×10^-7^, interaction p=2.8×10^-5^). **(F, G)** The density and size of the VGluT1^+^ excitatory and VGAT^+^ inhibitory terminals in the SR of the vCA1 were compared between X-ray (n=6) and X-ray+ECS (n=6). **(H)** The percent area of the PLP^+^ myelin in the SR was compared between X-ray (n=6) and X-ray+ECS (n=6). * p<0.05 (Student’s t-test, vs. CTL).


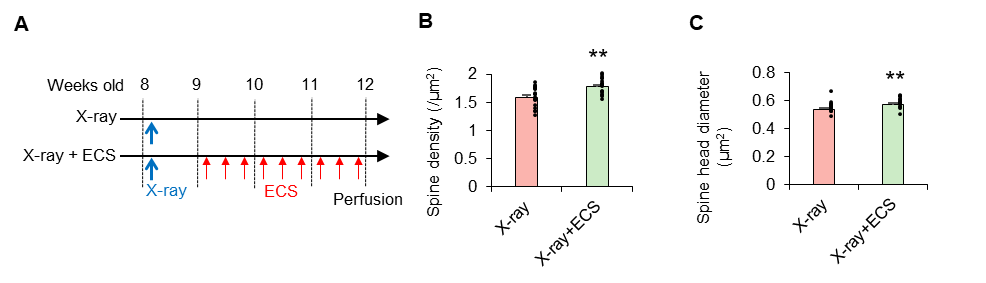


**Supplemental Fig. 13. ECS increases the density and size of the spines in the vCA1 regardless of the presence of neurogenesis.**

**(A)** The time course of ECS and X-ray irradiation. **(B, C)** The spine density and size from the Golgi stain were compared in the SR of the vCA1 between X-ray and X-ray+ECS (20 neurons were counted from three mice of each group). ** p<0.01, (Student’s t-test, vs. CTL).


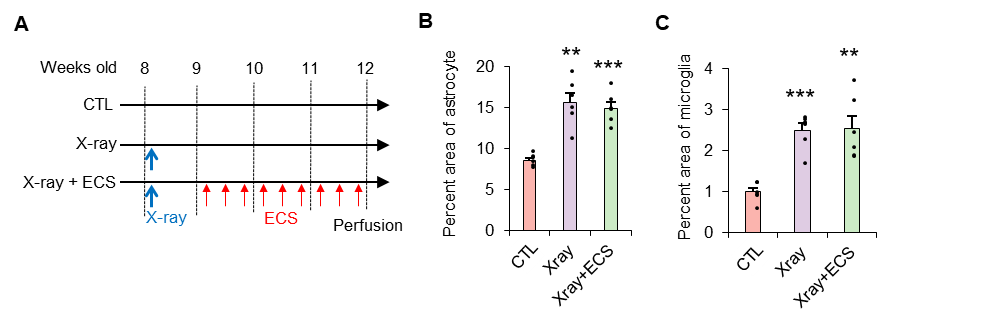


**Supplemental Fig. 14. Glial activation is induced by X-ray irradiation in the vCA1.**

**(A)** The time course of ECS and X-ray irradiation. **(B, C)** The percent area of the GLT1^+^ astrocytes and Iba1^+^ microglia were compared in the SR of the vCA1 among CTL (n=6), X-ray (n=6), and X-ray+ECS (n=6). ** p<0.01, *** p<0.001 (Student’s t-test, p-values were Bonferroni corrected, vs. CTL).

| **Regions** | **Sides** | **Volume (mm^3^) of dose-dependent experiments** | | | |  | **Volume (mm^3^) of X-ray irradiation experiments** | | | |
| --- | --- | --- | --- | --- | --- | --- | --- | --- | --- | --- |
|  |  | **CTL** | **3×ECS** | **6×ECS** | **9×ECS** |  | **CTL** | **9×ECS** | **Xray** | **Xray+9×ECS** |
| Whole hippocampus | Left | 7.63±0.13 | 7.48±0.09 | 7.47±0.08 | 8.24±0.13*** |  | 7.41±0.12 | 7.99±0.13** | 7.00±0.09 | 7.81±0.13^##^ |
|  | Right | 7.76±0.12 | 7.59±0.08 | 7.59±0.09 | 8.30±0.13*** |  | 7.47±0.11 | 8.04±0.11** | 7.12±0.03 | 7.87±0.08^##^ |
| Dorsal hippocampus | Left | 3.77±0.09 | 3.69±0.06 | 3.63±0.04 | 4.09±0.07** |  | 3.43±0.08 | 3.72±0.06* | 3.22±0.06 | 4.09±0.07^##^ |
|  | Right | 3.87±0.08 | 3.77±0.05 | 3.73±0.05 | 4.13±0.06** |  | 3.52±0.07 | 3.79±0.06* | 3.29±0.06 | 3.68±0.05^##^ |
| Ventral hippocampus | Left | 3.85±0.05 | 3.77±0.04 | 3.82±0.05 | 4.13±0.06*** |  | 3.98±0.04 | 4.27±0.07** | 3.78±0.05 | 4.18±0.07^##^ |
|  | Right | 3.86±0.05 | 3.83±0.03 | 3.84±0.05 | 4.16±0.06*** |  | 3.94±0.05 | 4.25±0.06** | 3.83±0.05 | 4.19±0.04^##^ |
| DG | Left | 1.61±0.03 | 1.58±0.02 | 1.56±0.02 | 1.73±0.02** |  | 2.08±0.04 | 2.24±0.04** | 1.95±0.03 | 2.18±0.04^##^ |
|  | Right | 1.68±0.03 | 1.65±0.02 | 1.63±0.02 | 1.80±0.02** |  | 2.11±0.04 | 2.28±0.03** | 1.99±0.03 | 2.21±0.03^##^ |
| CA3 | Left | 1.92±0.03 | 1.86±0.03 | 1.86±0.02 | 2.07±0.04* |  | 1.86±0.03 | 1.99±0.04 | 1.74±0.03 | 1.93±0.03^#^ |
|  | Right | 1.89±0.04 | 1.83±0.02 | 1.80±0.02 | 1.99±0.03* |  | 1.86±0.03 | 1.97±0.03 | 1.76±0.03 | 1.91±0.02^#^ |
| CA2 | Left | 0.17±0.003 | 0.16±0.002 | 0.16±0.002 | 0.18±0.003* |  | 0.14±0.003 | 0.15±0.003 | 0.13±0.002 | 0.15±0.002^#^ |
|  | Right | 0.17±0.003 | 0.17±0.002 | 0.17±0.002 | 0.18±0.003* |  | 0.14±0.002 | 0.15±0.002 | 0.13±0.002 | 0.14±0.001^#^ |
| CA1 | Left | 3.67±0.06 | 3.63±0.04 | 3.63±0.04 | 3.96±0.06*** |  | 3.33±0.05 | 3.60±0.05** | 3.18±0.04 | 3.54±0.06^##^ |
|  | Right | 3.71±0.06 | 3.70±0.04 | 3.64±0.05 | 4.02±0.05*** |  | 3.36±0.05 | 3.765±0.05** | 3.23±0.04 | 3.60±0.04^##^ |

**Supplementary Table 1. An ROI-based analysis of brain volume changes in the whole hippocampus, the dorsal and ventral segments, and the hippocampal subfields.**

The volumes (mm^3^, Mean±SEM) of each hippocampal region are summarized for the dose-dependent and X-ray irradiation experiments, respectively. *p<0.05, **p<0.01, ***p<0.001 (Student’s t-test; p-values were Bonferroni-corrected; comparisons with the CTL group). # p<0.05, ## p<0.01 (Student’s t-test; p-values were Bonferroni-corrected; comparisons with the X-ray group).

| Histological assessments | Target area | CTL | 3×ECS | 6×ECS | 9×ECS | correlation with MRI |  |
| --- | --- | --- | --- | --- | --- | --- | --- |
|  |  |  |  |  |  | Peason's r | p-value |
| VGluT1 density (/μm^2^) | vCA1 SR layer | 0.312±0.009 | 0.426±0.019** | 0.487±0.001** | 0.532±0.016*** | 0.67 | <0.001 |
|  | vCA1 SO layer | 0.443±0.023 | - | - | 0.589±0.041* | - | - |
|  | vCA1 SLM layer | 0.446±0.026 | - | - | 0.437±0.029 | - | - |
|  | vDG | 0.595±0.026 | - | - | 0.427±0.022*** | - | - |
| VGluT1 area (μm2) | vCA1 SR layer | 0.114±0.009 | 0.115±0.007 | 0.111±0.004 | 0.115±0.004 | 0.06 | 0.73 |
|  | vCA1 SO layer | 0.142±0.005 | - | - | 0.141±0.004 | - | - |
|  | vCA1 SLM layer | 0.144±0.007 | - | - | 0.140±0.003 | - | - |
|  | vDG | 0.108±0.007 | - | - | 0.114±0.009 | - | - |
| Synaptic (PSD95/VGluT1) density (/μm2) | vCA1 SR layer | 0.584±0.034 | - | - | 1.094±0.104* | - | - |
|  | vCA1 SO layer | 0.853±0.081 | - | - | 1.178±0.081* | - | - |
|  | vCA1 SLM layer | 0.791±0.046 | - | - | 0.900±0.048 | - | - |
| VGAT density (/μm2) | vCA1 SR layer | 0.095±0.006 | 0.085±0.003 | 0.107±0.007 | 0.135±0.006** | 0.55 | <0.001 |
|  | vCA1 SO layer | 0.117±0.008 | - | - | 0.134±0.008 | - | - |
|  | vCA1 SLM layer | 0.152±0.008 | - | - | 0.158±0.009 | - | - |
|  | vDG | 0.155±0.011 | - | - | 0.154±0.010 | - | - |
| VGAT area (μm2) | vCA1 SR layer | 0.192±0.007 | 0.158±0.005** | 0.179±0.004 | 0.227±0.008** | 0.39 | <0.001 |
|  | vCA1 SO layer | 0.178±0.011 | - | - | 0.231±0.013* | - | - |
|  | vCA1 SLM layer | 0.178±0.011 | - | - | 0.198±0.008 | - | - |
|  | vDG | 0.213±0.006 | - | - | 0.198±0.004 | - | - |
| % area of PLP (mylination) | vCA1 SR layer | 1.332±0.129 | 1.058±0.067 | 1.879±0.088** | 0.235±0.161*** | 0.47 | 0.004 |
|  | vCA1 SO layer | 2.783±0.214 | - | - | 2.917±0.181 | - | - |
|  | vCA1 SLM layer | 3.414±0.245 | - | - | 3.304±0.309 | - | - |

**Supplemental Table 2. A summary of the histological values and correlations with MRI data.**

Each histological value is listed for 4 groups (CTL, 3×ECS, 6×ECS, 9×ECS). Peason’s r values and p-values are also listed for the correlation between each histological value and significant cluster values of the MRI regression analysis.
